# Natural history of a mouse model of X-linked myotubular myopathy

**DOI:** 10.1242/dmm.049342

**Published:** 2022-07-25

**Authors:** Ege Sarikaya, Nesrin Sabha, Jonathan Volpatti, Emanuela Pannia, Nika Maani, Hernan D. Gonorazky, Alper Celik, Yijng Liang, Paula Onofre-Oliveira, James J. Dowling

**Affiliations:** 1 Program for Genetics and Genome Biology, The Hospital for Sick Children, 686 Bay Street, Toronto, ON M5G 1X8, Canada; 2 Department of Molecular Genetics, University of Toronto, 1 King's College Circle, Toronto, ON M5S 1A8, Canada; 3 Division of Neurology, The Hospital for Sick Children, 686 Bay Street, Toronto, ON M5G 1X8, Canada; 4 Centre for Computational Medicine, The Hospital for Sick Children, 686 Bay Street, Toronto, ON M5G 1X8, Canada; 5 Paediatrics, University of Toronto, 1 King's College Circle, Toronto, ON M5S 1A8, Canada

**Keywords:** Mice, Myotubularin, Myotubular myopathy, Muscle disease, Natural history

## Abstract

X-linked myotubular myopathy (XLMTM) is a severe monogenetic disorder of the skeletal muscle. It is caused by loss-of-expression/function mutations in the myotubularin (*MTM1*) gene. Much of what is known about the disease, as well as the treatment strategies, has been uncovered through experimentation in pre-clinical models, particularly the *Mtm1* gene knockout mouse line (*Mtm1* KO). Despite this understanding, and the identification of potential therapies, much remains to be understood about XLMTM disease pathomechanisms, and about the normal functions of MTM1 in muscle development. To lay the groundwork for addressing these knowledge gaps, we performed a natural history study of *Mtm1* KO mice. This included longitudinal comparative analyses of motor phenotype, transcriptome and proteome profiles, muscle structure and targeted molecular pathways. We identified age-associated changes in gene expression, mitochondrial function, myofiber size and key molecular markers, including DNM2. Importantly, some molecular and histopathologic changes preceded overt phenotypic changes, while others, such as triad structural alternations, occurred coincidentally with the presence of severe weakness. In total, this study provides a comprehensive longitudinal evaluation of the murine XLMTM disease process, and thus provides a critical framework for future investigations.

## Abstract (precis)

**Summary:** This study provides a comprehensive and longitudinal molecular and phenotypic evaluation of the disease process of X-linked myotubular myopathy (XLMTM) in a murine model.

## INTRODUCTION

X-linked myotubular myopathy (XLMTM) is a monogenetic muscle disease with onset in infancy, characterized by neonatal hypotonia and severe weakness (Lawlor and Dowling, 2021). Between 25% and 50% of affected patients die in the first year of life, and those that survive have a high degree of technology dependence (80% requiring wheelchair, ventilator and feeding tube support) and shortened life span (Amburgey et al., 2017; Annoussamy et al., 2019; Beggs et al., 2018). Historically, the condition was diagnosed by characteristic features on muscle biopsy (centrally located myonuclei, myofiber hypotrophy, and accumulation of mitochondria and other perinuclear organelles); currently, diagnosis is established with positive genetic testing (Gonorazky et al., 2018).

XLMTM is caused by mutations in myotubularin (*MTM1*) that result in loss of expression and/or function of the MTM1 protein. MTM1 is a lipid phosphatase that acts on phosphoinositides (PIPs) (Laporte et al., 1996; Raess et al., 2017); specifically, it dephosphorylates (and thus deactivates) PI3P and PI3,5P2, and through this action also generates PI5P (Blondeau et al., 2000; Taylor et al., 2000; Volpatti et al., 2019). MTM1 is a resident endosomal protein, and its main role in cells is to regulate vesicular sorting through the endosomal compartment via a PIP conversion mechanism (Ketel et al., 2016).

Several pathologic changes have been identified in muscle from *MTM1*-deficient patients and animal models. In particular, abnormalities in the appearance and function of the excitation–contraction (EC) coupling apparatus have been observed (Al-Qusairi et al., 2009; Dowling et al., 2009). EC coupling is the process via which signals initiated at the neuromuscular junction are converted to muscle contraction (Dowling et al., 2021). The key muscle substructure that mediates this process is the triad, which represents the apposition of the T-tubule and the terminal sarcoplasmic reticulum. In XLMTM, both triad structure and EC coupling are abnormal, and are thought to be responsible for the weakness observed in patients (Jungbluth et al., 2018).

Several molecular abnormalities have also been described. These include changes in the AKT pathway, aggregation of desmin intermediate filaments (Hnia et al., 2011), increased levels of the large GTPase DNM2 (Cowling et al., 2014), altered protein ubiquitination (Gavriilidis et al., 2018) and changes in autophagic flux (Al-Qusairi et al., 2013; Bachmann et al., 2017; Fetalvero et al., 2013). Perhaps the most important of these is the upregulation of DNM2 protein expression, as this has been shown to directly lead to many of the pathologic changes characteristic of XLMTM (Cowling et al., 2011; Liu et al., 2011). In addition, therapies aimed at lowering DNM2 (including antisense oligonucleotide-mediated knockdown and chemical reduction with tamoxifen) have shown great efficacy in pre-clinical models and are currently in clinical trial in patients (Buono et al., 2018; Gayi et al., 2018; Maani et al., 2018; Tasfaout et al., 2017). Of note, gene replacement therapy can rescue the phenotype of pre-clinical models and is also in clinical trial (Childers et al., 2014).

Much of what is known about XLMTM has been uncovered through experimentation using an *Mtm1* mouse knockout line (*Mtm1* KO). The model, first generated by Buj-Bello and Laporte (Buj-Bello et al., 2002), has a targeted deletion in exon 4 of *Mtm1*, resulting in a frameshift and stop gain, reduction in *Mtm1* mRNA expression and absence of MTM1 protein expression. It was originally created on the 129S2PAS background, and subsequently has been outcrossed to other strains including C57BL/6J. Other animal models have also been established, such as XLMTM zebrafish and canine models (Beggs et al., 2010; Dowling et al., 2009; Sabha et al., 2016). All vertebrate models tested thus far faithfully recapitulate the major abnormalities seen in patient muscle, and are associated with abnormal motor function and early lethality.

Although animal models have significantly advanced our understanding of XLMTM, much remains to be understood about the underlying pathomechanisms of XLMTM. In particular, the link between the role of MTM1 as a phosphoinositide phosphatase and regulator of endosomal dynamics, and the pathologic and molecular changes in XLMTM muscle are not clear. Furthermore, it is not known what the first/inciting events are in the disease process. As more therapies are identified for the disease, it is critical to understand how these treatments may synergize, and how they compare in terms of effectiveness for ameliorating key aspects of the disease.

To establish the required groundwork for addressing these key unknowns, we have undertaken to define the natural history of the *Mtm1* KO mice. By evaluating pathologic and molecular events at different time points, we have identified that mitochondrial abnormalities, widespread transcriptional changes, myofiber hypotrophy and increased DNM2 are among the earliest changes observed. Surprisingly, triad changes are found later in the disease process, after a period of normal triad appearance, and coincide with severe phenotypic alterations. In total, our work provides critical longitudinal data important for future studies related to therapy testing and the establishment of disease pathomechanisms.

## RESULTS

### *Mtm1* KO mice have four phenotypic stages of disease

**Fig. 1.**
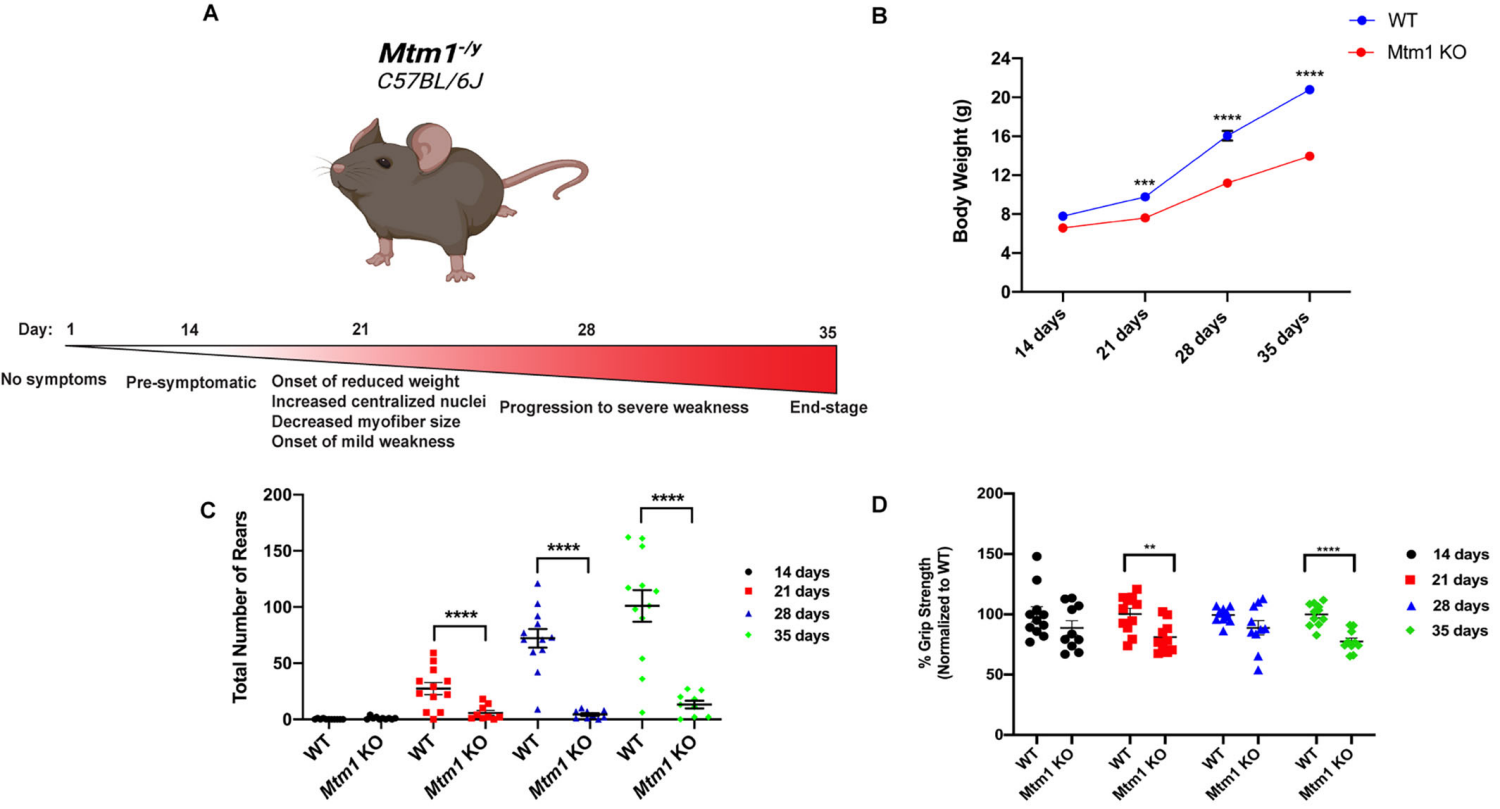
**Phenotypic studies of the *Mtm1* knockout mouse model.** (A) Schematic depicting the four phases of the disease process in *Mtm1* knockout (*Mtm1^−/y^* or *Mtm1* KO) mice. (B-D) Longitudinal phenotypic analyses of wild-type (WT) littermates versus *Mtm1* KO mice at 14, 21, 28 and 35 days (WT *n*=11, KO *n*=10). (B) Scatter plot comparing weight (g) of WT versus *Mtm1* KO mice (values are mean±s.e.m.). 14 days: WT 7.8±0.3 g, KO 6.6±0.3 g; 21 days: WT 9.8±0.3 g, KO 7.6±0.4 g (***P<0.001); 28 days: WT 16.0±0.5 g, KO 11.2±0.3 g (****P<0.0001); and 35 days: WT 21.0±0.5 g, KO 14.0±0.4 g (****P<0.0001). (C) Number of rears (as measured by open field testing; values are mean±s.e.m.). 14 days: WT 0.2±0.1, KO 1±0.4; 21 days: WT 27.4±5.3, KO 5.8±2.2 (****P<0.0001), 28 days: WT 72.2±8.2, KO 4.4±1.2 (****P<0.0001); and 35 days: WT 101±14.2, KO 13.2±3.4 (****P<0.0001). (D) Grip strength (expressed as a percentage of WT; values are mean±s.e.m.). 14 days: WT 100±6.3%, KO 88.8±6%; 21 days: 100.3±4.6%, KO 81.1±3.9% (**P<0.001); 28 days: WT 99.4±1.8%, KO 88.8±6%; and 35 days: WT 100±2.8%, KO 77.5±3% (****P<0.0001). Statistical analysis by log-rank (Mantel–Cox) test or unpaired two-tailed Student's *t*-test.

We have previously established a colony of *Mtm1* KO mice on the C57BL/6J background (Maani et al., 2018; Sabha et al., 2016). In this study, we performed an extensive phenotypic and molecular characterization of this mouse line. For phenotypic assessment, we examined body weight, grip strength and motor behavior via open field testing (Fig. 1). This was done serially on the same set of mice. Based on this, we observed four phases of disease (Fig. 1A). The first is the pre-symptomatic phase, which occurs until the age of weaning [postnatal day (P)21]. For analysis purposes, we have chosen 14 days to represent the first time period. The second is symptom onset, which occurs at ∼P21. The first observed phenotypic abnormalities at this time are lower body weight and reduced animal rears (Fig. 1B,C). The third is onset of severe motor deficits, which in our hands occurs at ∼28 days of age. This is characterized in open field testing by both a near complete inability to rear as well as ambulatory defects, including reductions in the total distance traveled and average travel speed (Fig. S1). The last is the endpoint phase, at which *Mtm1* KO mice have severe hindlimb paralysis, as evidenced by a large reduction in grip strength (Fig. 1D) and generalized wasting, and typically require termination. This occurs at 35 days and beyond (median survival 38 days) (Maani et al., 2018; Sabha et al., 2016).

### Mitochondrial and sarcolemmal disorganization represent the first structural changes

**Fig. 2.**
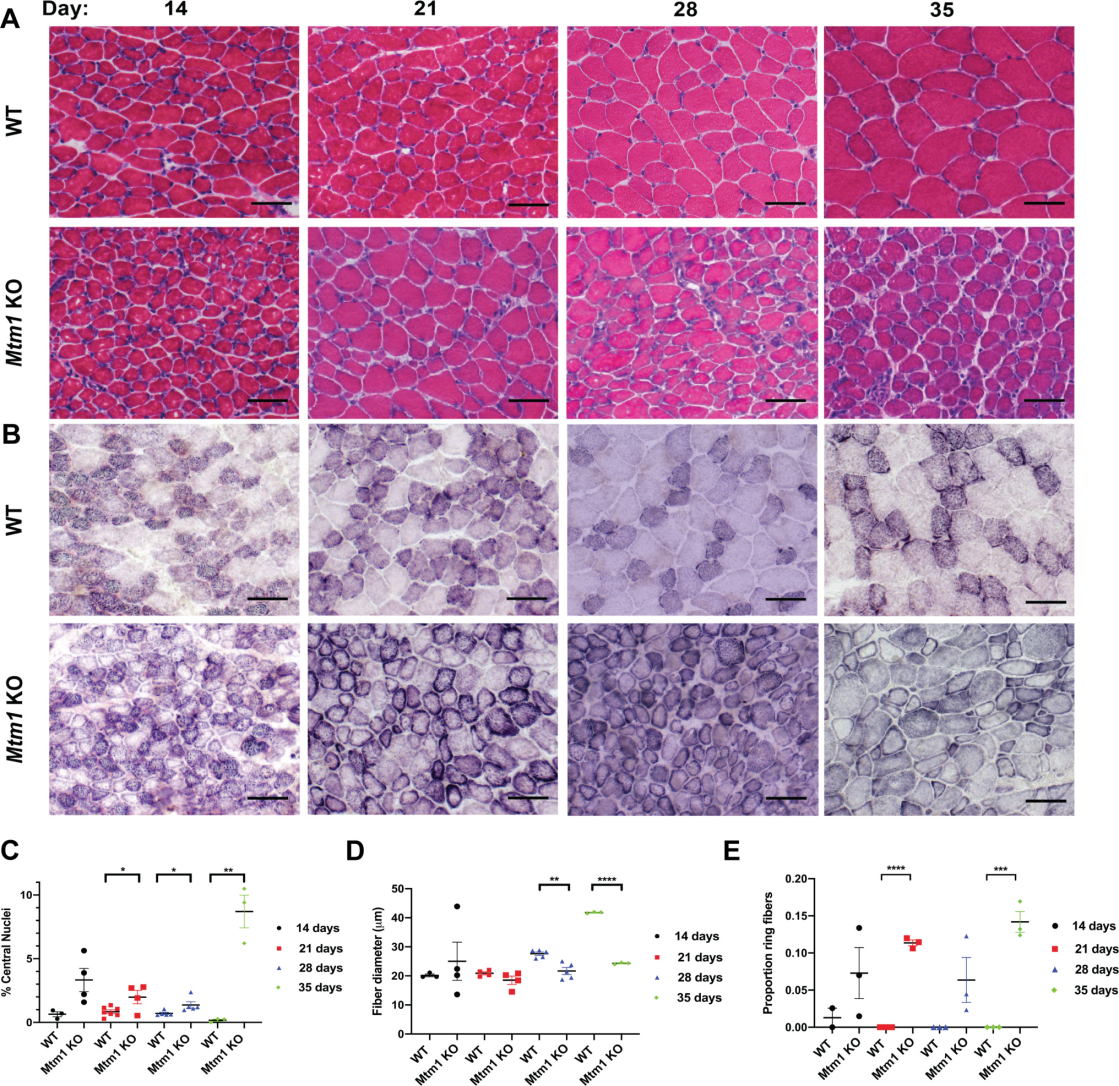
**Progressive histological changes in *Mtm1* knockout mice.** (A,B) Histopathology as defined by staining with Hematoxylin and Eosin (H&E) (A) and succinate dehydrogenase (SDH) (B). Scale bars: 50 µm. (C) There is an observable increase in % central nuclei in *Mtm1* KO mice compared to WT starting at 21 days (WT *n*=3-7, KO *n*=4 per time point). % central nuclei (values are mean±s.e.m.). 14 days: WT 0.64±0.20%, KO: 3.32±0.90%; 21 days: WT 0.84±0.14%, KO 1.98±0.52% (*P<0.05); 28 days: WT 0.70±0.10%, KO 1.37±0.26% (*P<0.05); and 35 days: WT 0.17±0.09%, KO 8.70±1.3% (**P<0.01). (D) *Mtm1* KO mice have significantly smaller fibers starting at 28 days. Fiber size (WT *n*=3-5, KO *n*=3-5; values are mean±s.e.m.). 14 days: WT 20.16±0.47 μm, KO 25.03±6.57 μm; 21 days: WT 20.89±0.51 μm, KO 18.51±1.43 μm; 28 days: WT 27.66±0.59 μm, KO 21.67±1.21 μm (**P<0.001); and 35 days: WT 46.02±3.77 μm, KO 21.78±0.47 μm (****P<0.0001). (E) Quantification of ring fibers, as determined with SDH staining (WT *n*=2-3, KO *n*=3 per time point; values are mean±s.e.m.). 14 days: WT 0.01±0.01, KO 0.072±0.034; 21 days: WT 0, KO 0.11±0 (****P<0.0001); 28 days: WT 0, KO 0.063±0.03; and 35 days WT 0, KO 0.14±0 (***P<0.001). Statistical analysis by unpaired two-tailed Student's *t*-test.

**Fig. 3.**
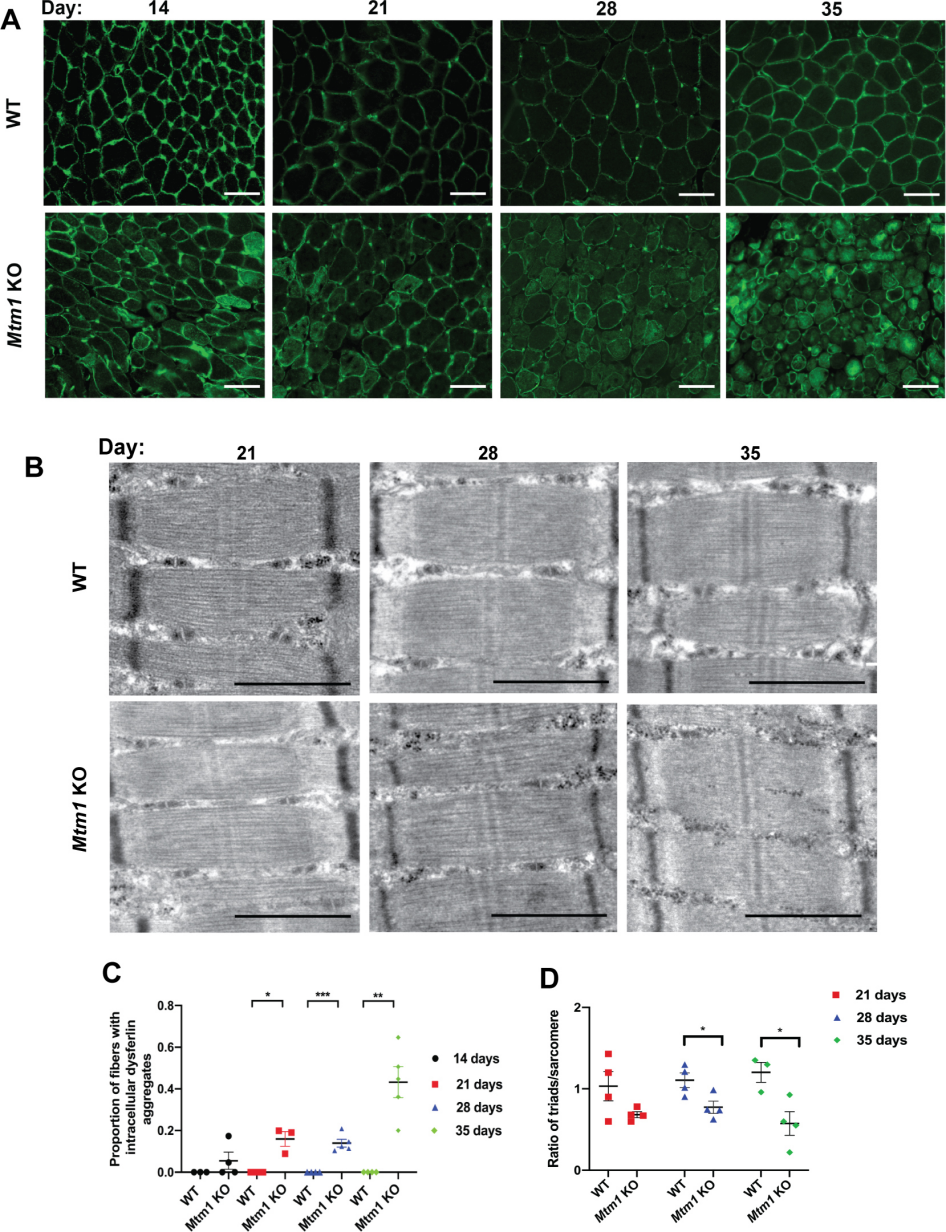
**Progressive histopathologic and ultrastructural changes in *Mtm1* knockout mice.** (A) Dysferlin immunofluorescence at 14, 21, 28 and 35 days on skeletal muscle from WT and *Mtm1* KO mice. Scale bars: 50 µm. (B) Electron microscopy of skeletal muscle from WT and *Mtm1* KO mice. Scale bars: 1 µm. Ratios of triads/sarcomere (WT *n*=3-4, KO *n*=4; values are mean±s.e.m.). 21 days: WT 1.03±0.18, KO 0.68±0.04; 28 days: WT 1.11±0.10, KO 0.77±0.10 (*P<0.05); and 35 days: WT 1.20±0.12, KO 0.57±0.14 (*P<0.05). (C) Quantification of dysferlin immunostaining. An increasing proportion of myofibers with intracellular dysferlin aggregates is observed in *Mtm1* KO mice starting at 21 days. Proportion of myofibers with intracellular dysferlin aggregations (WT *n*=3-7, KO *n*=4 per time point; values are mean±s.e.m.). 14 days: WT 0, KO 0.055±0.04; 21 days: WT 0, KO 0.16±0.04 (*P<0.05); 28 days: WT 0, KO 0.14±0.02 (***P<0.001); and 35 days: WT 0, KO 0.43±0.07 (**P<0.001). (D) Quantification of triad number. Triad number per sarcomere (WT *n*=3-4, KO *n*=4 per time point; values are mean±s.e.m.). 21 days: WT 1.1±0.11, KO 0.68±0.18; 28 days: WT 1.2±0.19, KO 0.77±0.11 (*P<0.05); and 35 days: WT 1.30±0.31, KO 0.57±0.19 (*P<0.05). Statistical analysis by unpaired two-tailed Student's *t*-test.

We next analyzed muscle histopathology at the four defined stages (Figs 2 and 3). We specifically evaluated changes that have been previously described in *Mtm1* KO mice, which include % central nuclei and myofiber size [both quantified from Hematoxylin and Eosin (H&E) staining], mitochondrial distribution [defined by succinate dehydrogenase staining (SDH)], sarcolemmal membrane organization (illuminated by dysferlin immunostaining), and triad structure (defined by electron microscopy). We performed these analyses on cryosections of skeletal muscle [tibialis anterior (TA)] at P14, P21, P28 and P35 in *Mtm1* KO versus wild-type (WT) littermates, using a minimum *n*=4 per genotype with blinded examination.

Interestingly, despite the fact that mice are overtly normal at 14 days of age, muscle structure was clearly altered at this stage (Fig. 2A). The most striking finding was redistribution of mitochondria into ‘ring’ fibers, something that was seen on occasion in WT at 14 days, but that was significantly increased in *Mtm1* KO mice (average of 1.0 per field for WT versus 6.7 for *Mtm1* KO). There were also small but significant changes in % central nuclei and dysferlin immunolocalization. Although the distribution of fiber size was altered, mean myofiber diameter was not significantly changed. It is notable that none of the changes occurred in more than a minority of fibers.

At 21 days, these abnormalities were more pronounced (Fig. 2A,B), but triad structure was unchanged (Fig. 3B). In fact, there was no quantitative difference in the number or appearance of triads at this age. It is not until 28 days of age that triads became statistically different in *Mtm1* KO versus WT (Fig. 3B). By 35 days of age, there were very few muscle fibers that appeared normal, and a near complete loss of triads.

### Muscles are differentially affected in *Mtm1* KO mice

**Fig. 4.**
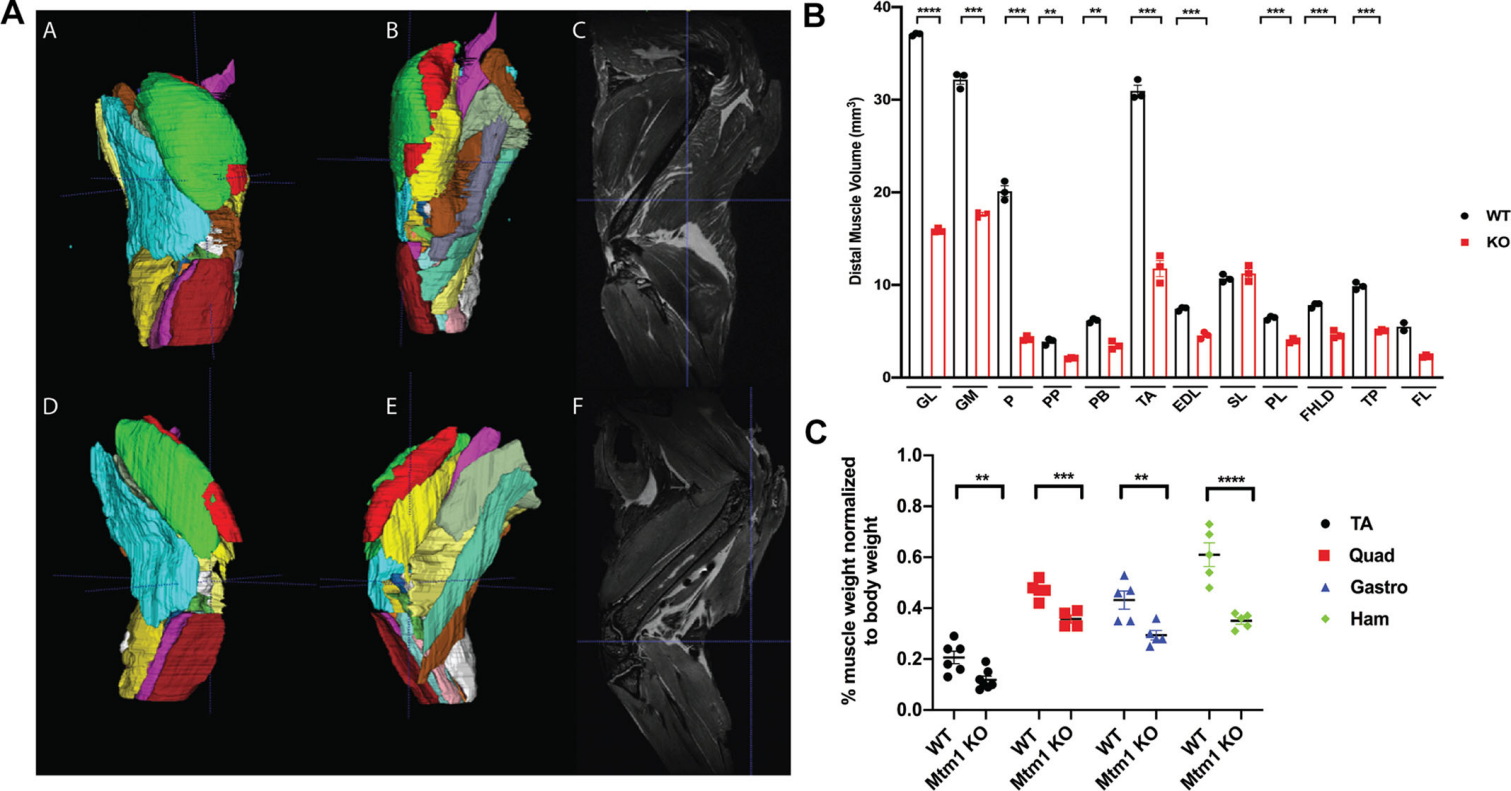
**Skeletal muscle magnetic resonance imaging (MRI) of WT and *Mtm1* knockout mice.** (A) T2-weighted muscle MRI images from immediately post-mortem hindlimbs of WT and *Mtm1* KO mice performed at 35 days. Segmentation was done manually using ITK-SNAP software. (B) Graph depicting distal muscle volume for 12 skeletal muscles as determined from MRI (WT *n*=3, *Mtm1* KO *n*=3; values are mean±s.e.m.). Distal muscle volume (mm^3^) for GL: WT 37.07±0.09 mm^3^, KO 15.9±0.13 mm^3^ (****P<0.0001); GM: WT 32.18±0.51 mm^3^, KO 17.62±0.20 mm^3^ (***P<0.001); P: WT 20.12±0.60 mm^3^, KO 4.2±0.16 mm^3^ (***P<0.001); PP: WT 3.90±0.20 mm^3^, KO 2.08±0.05 mm^3^ (**P<0.001); PB: WT 6.15±0.13 mm^3^, KO 3.47±0.26 mm^3^ (**P<0.001); TA: WT 31.0±0.62 mm^3^, KO 11.78±0.86 mm^3^ (***P<0.001); EDL: WT 7.42±0.12 mm^3^, KO 4.60±0.21 mm^3^ (***P<0.001); SL: WT 10.7±0.25 mm^3^, KO 11.23±0.50 mm^3^; PL: WT 6.43±0.12 mm^3^, KO 4.0±0.13 mm^3^ (***P<0.001); FHLD: WT 7.82±0. 16 mm^3^, KO 4.62±0.25 mm^3^ (***P<0.001); TP: WT 9.90±0.23 mm^3^, KO 5.10±0.08 mm^3^ (***P<0.001); FL: WT 5.50±0.43 mm^3^, KO 2.30±0.08 mm^3^. *n*=3 per condition. GL, gastrocnemius lateralis, GM, gastrocnemius medialis, P, plantaris; PP, popliteus; PB, peroneus brevis; TA, tibialis anterior; EDL, extensor digitalis longus; SL, soleus; PL, peroneus longus; FHLD, flexor hallucis longus; TP, tibialis posterior; FL, flexor digitorum longus. (C) Muscle weight (expressed as a percentage of total body weight muscle weight for TA, Quad, Gastro and Ham) normalized to body weight at 35 days (WT *n*=4-6, KO *n*=4-6; values are mean±s.e.m.). Percentage weight for TA: WT 0.20±0.02%, KO 0.11±0.01% (**P<0.001); Quad: WT 0.47±0.02%, KO 0.36±0.02% (***P<0.001); Gastro: WT 0.43±0.04%, KO 0.30±0.02% (**P<0.001); and Ham: WT 0.61±0.46%, KO 0.35±0.13% (****P<0.0001). TA, tibialis anterior; Quad, quadriceps; Gastro, gastrocnemius; Ham, hamstring. Statistical analysis by unpaired two-tailed Student's *t*-test.

**Fig. 5.**
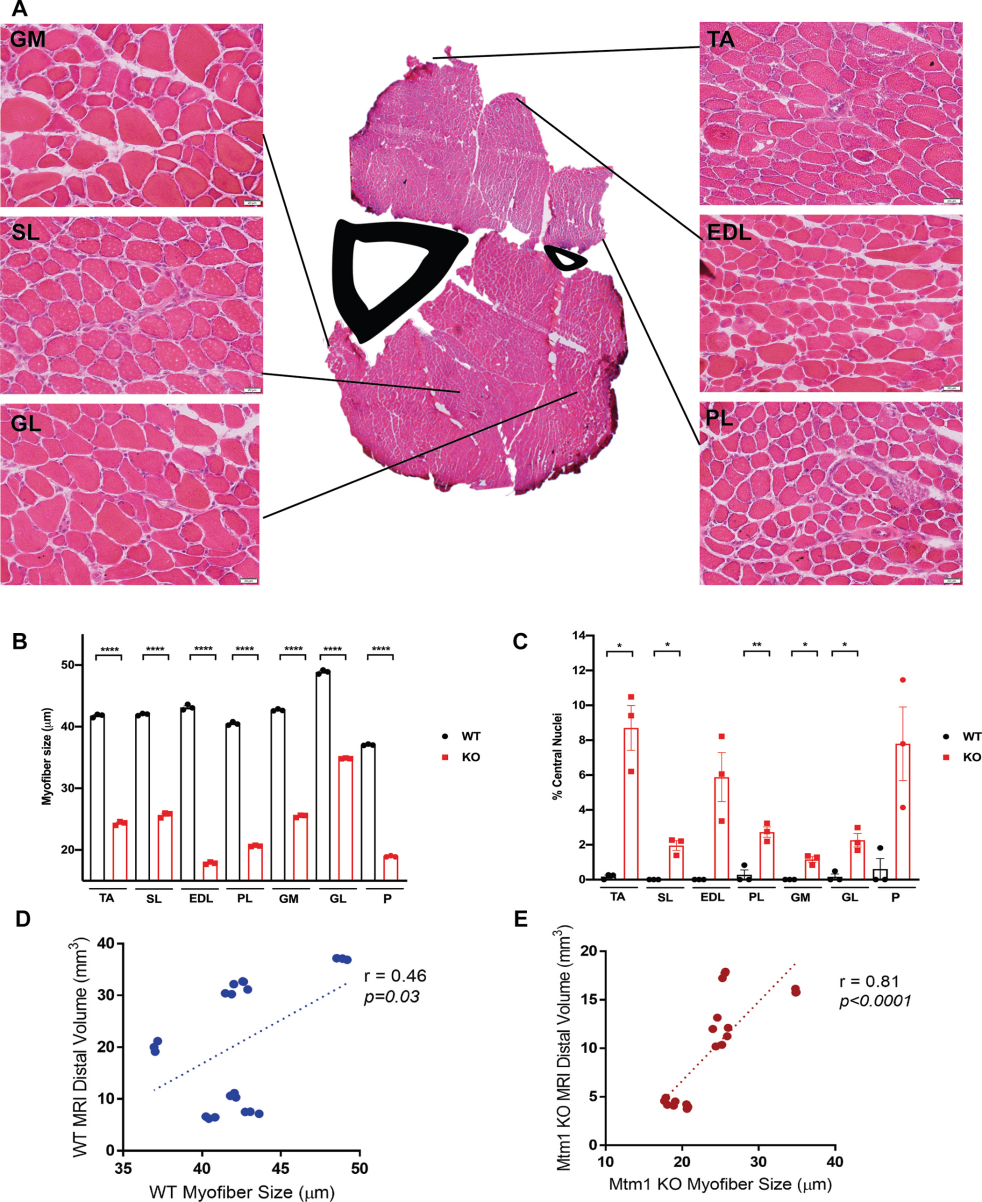
**Comparison of histopathologic characteristics between muscle groups.** (A) Representative images from H&E-stained muscle cryosections from the distal portion of the hindlimb from 35-day-old *Mtm1* KO mice (WT *n*=3, KO *n*=3 for each). Scale bars: 20 µm. (B,C) Graphs depicting myofiber size and % central nuclei for individual skeletal muscles of the hindlimb of WT and *Mtm1* KO mice at 35 days. (B) Myofiber size (μm; values are mean±s.e.m.). TA: WT 41.78±0.16 μm, KO 24.33±0.18 μm (****P<0.0001); SL: WT 42±0.10 μm, KO 25.70±0.25 μm (****P<0.0001); EDL: WT 43.14±0.26 μm, KO 17.86±0.11 μm (****P<0.0001); PL: WT 40.50±0.16 μm, KO 20.65±0.06 μm (****P<0.0001); GM: WT 42.70±0.10 μm, KO 25.50±0.11 μm (****P<0.0001); GL: WT 48.90±0.19 μm, KO 34.85±0.04 μm (****P<0.0001); P: WT 37.06±0.07 μm, KO 19.0±0.07 μm (****P<0.0001). (C) % central nuclei (values are mean±s.e.m.). TA: WT 0.17±0.09%, KO 8.7±1.3% (*P<0.05); SL: WT 0%, KO 2.0±0.3% (*P<0.05); EDL: WT 0%, KO 5.9±1.4%; PL: WT 0.28±0.28%, KO 2.8±0.3% (**P<0.01), GM: WT 0%, KO 1.2±0.2% (*P<0.05), GL: WT 0.16±0.16%, KO 2.3±0.40% (*P<0.05); P: WT 0.61±0.61%, KO 7.8±2.1%. TA, tibialis anterior; SL, soleus; EDL, extensor digitalis longus; PL, peroneus longus; GM, gastrocnemius medialis; GL, gastrocnemius lateralis; P, plantaris. Statistical analysis by unpaired two-tailed Student's *t*-test. (D,E) Pearson correlation coefficient (*r*) derived from linear regression of myofiber size (diameter, μm) and MRI distal volume (mm^3^) (Fig. 4B) in WT (D) and *Mtm1* KO (E) mice at 35 days. Significance indicated at *P*<0.05.

In XLMTM, there was variability in the extent of pathology seen in different muscle groups. This is best appreciated in patients by using muscle magnetic resonance imaging (MRI) (Carlier and Quijano-Roy, 2019). We examined this in our *Mtm1* KO mice, using both MRI and matched histopathological analysis. We studied mice at 35 days of age, which was the earliest tractable age for MRI scanning in terms of mouse size. Using T2-weighted MRI images, we defined muscle volume of the muscle in the distal hindlimb (Fig. 4). We found that plantaris and TA were the most affected in terms of reduced size, while the soleus and tibialis posterior were the least affected (Fig. 4B). To relate these findings to standard muscle pathology, we measured myofiber size and % central nuclei from H&E-stained cryosections of muscle taken from the same mice that we imaged (Fig. 5A-C). Plantaris displayed the largest magnitude of change in myofiber size (Fig. 5B), with TA also being substantially decreased. We also saw a similar degree of changes between MRI findings and % central nuclei, with plantaris and TA having the highest % (Fig. 5C). Conversely, extensor digitorum longus (EDL), which was relatively mildly affected per MRI, had significant changes in myofiber size and % central nuclei. We further performed a correlation analysis between myofiber size and MRI volume of all muscles and found a positive correlation in both WT and *Mtm1* KO mice (Fig. 5D,E). Lastly, we examined muscle volume by measuring total muscle weight for a subset of muscles at 35 days of age (Fig. 4C). Muscle weight was reduced in *Mtm1* KO versus WT mice for all muscle groups examined, although the extent of change varied and matched that seen for these muscles on MRI.

### Increases in DNM2 protein levels and acetylated tubulin are among the first molecular changes observed in *Mtm1* KO mice

**Fig. 6.**
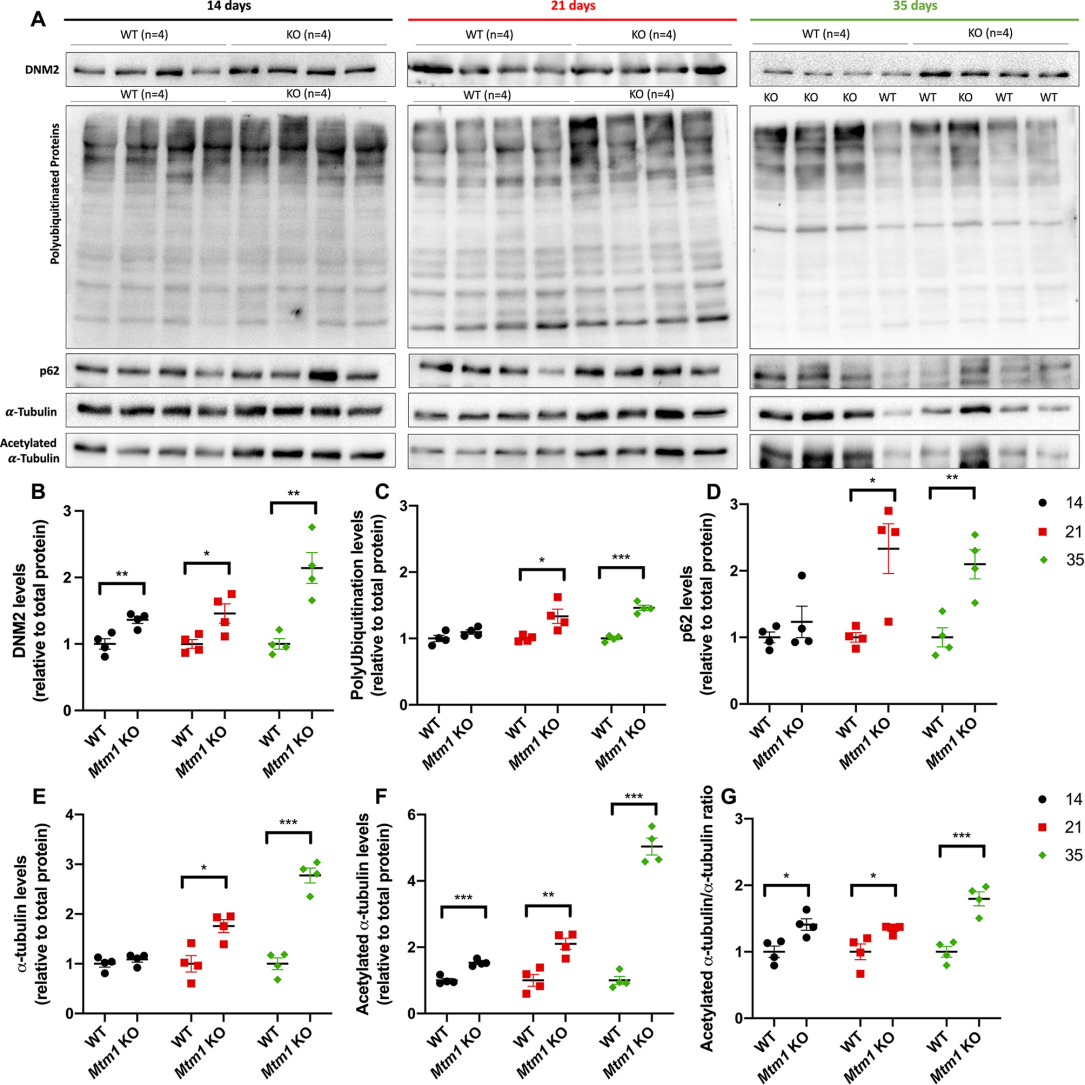
**Molecular changes in *Mtm1* KO mice.** (A) Representative western blot images of DNM2, polyubiquitinated protein, p62, α-tubulin and acetylated-α-tubulin from protein extracts of TA muscle taken at 14, 21 and 35 days. (B-G) Quantification of protein levels relative to total protein staining and normalized to average WT expression (see also Fig. S2). (B) DNM2 levels are elevated at 14 (1.36-fold, **P<0.01), 21 (1.46-fold, *P<0.05) and 35 (2.14-fold, **P<0.01) days. (C) Polyubiquitinated protein levels are elevated at 21 (1.33-fold, *P<0.05) and 35 (1.46-fold, ***P<0.001) days. (D) p62 levels are elevated at 21 (1.99-fold, *P<0.01) and 35 (2.10-fold, **P<0.01) days. (E) α-tubulin levels are elevated at 21 (1.76-fold, *P<0.05) and 35 (2.78-fold, ***P<0.001) days. (F) Acetylated-α-tubulin levels are elevated at 14 (1.53-fold, ***P<0.001), 21 (2.26-fold, **P=0.0041) and 35 (5.04-fold, ***P<0.001) days. (G) Acetylated-α-tubulin/α-tubulin ratio is elevated at 14 (1.41-fold, *P<0.05), 21 (1.34-fold, *P<0.05) and 35 (1.80-fold, ***P<0.001) days. All quantifications are from *n*=4 mice/genotype and done with three technical replicates. Statistical analysis by unpaired two-tailed Student's *t*-test.

We next examined by western blotting several of the molecular changes that have been described in *Mtm1* KO mice (Fig. 6). These included DNM2 levels, polyubiquitination, autophagy (as indicated by p62 levels) and acetylated tubulin. At P14, only DNM2 and tubulin were significantly changed in *Mtm1* KO compared to WT mice. The magnitude of change, however, was relatively small. At 21 days, all markers examined were abnormal, with an increased effect size in *Mtm1* KO versus WT at each age (Fig. 6).

### Comparative RNA sequencing (RNAseq) identifies emerging pathway abnormalities

**Fig. 7.**
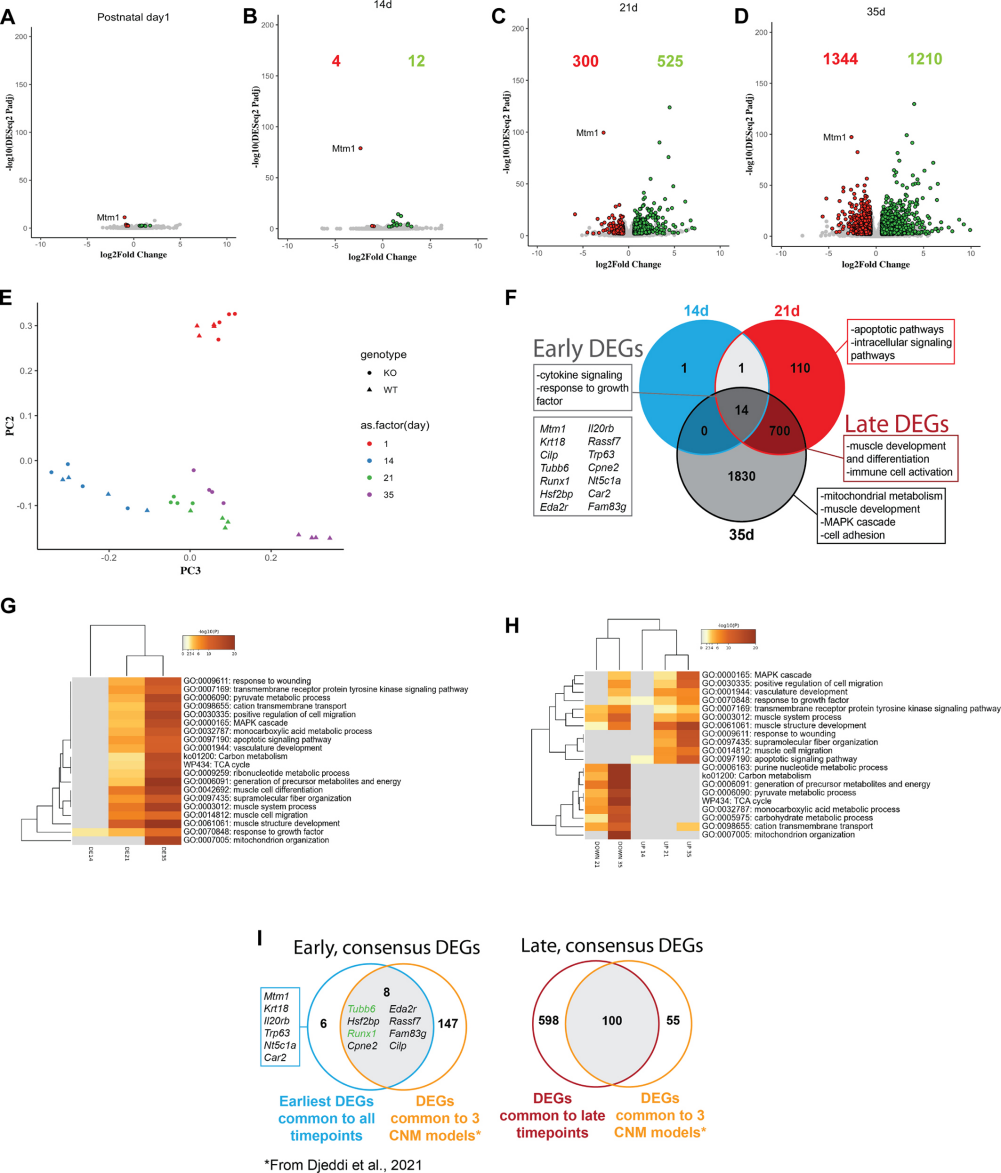
**Longitudinal transcription analysis of *Mtm1* knockout mice.** (A-D) Volcano plots highlighting differentially expressed genes (DEGs) between WT and *Mtm1* KO mice from TA muscle at 1 day (A), 14 days (B), 21 days (C) and 35 days (D) (adjusted *P*-value<0.01, log2FC>0.585). Statistical analysis by unpaired two-tailed Student's *t*-test. (E) Principal component analysis plot of all time points, indicating that gene expression is similar between WT and KO mice at the earliest stage tested but diverges starkly from 21 days onward. Note clustering of 21-day-old and 35-day-old KO mice, indicating shared transcriptional changes. (F) Venn diagram depicting common and unique DEGs at each time point. Boxes highlight major gene ontology (GO) enrichment terms with the associated DEG list. (G,H) Heat map visualization of GO terms enriched at each time point considering all DEGs in each time point (G) and by upregulated or downregulated status (H). (I) Venn diagram highlighting shared DEGs between our dataset and those identified as being in common in three centronuclear myopathy mouse models (including the *Mtm1* KO mice on a different background, 129S2PAS) by Djeddi et al. (2021).

To identify molecular pathways altered in *Mtm1* KO mice that associate with their defined phenotypes, we performed bulk RNAseq on TA at P1, P14, P21 and P35 (Fig. 7). Very few transcriptional changes were noted at 1 and 14 days of age, with only 16 differentially expressed genes (DEGs) at P14 (Fig. 7A,B). By 21 days, however, there were widespread alterations in gene expression, with more than 800 DEGs. By 35 days of age, >2500 DEGs were identified, with numerous cellular pathways disturbed compared to WT. Principal component analysis showed that the difference between genotypes increased with age (Fig. 7D). Furthermore, by principal component analysis, the transcriptomes of 35-day-old KO muscle segregated with those of 21-day-old KO muscle, suggesting shared features between these two aberrant profiles.

We considered which pathways were affected at each stage (beginning with P14) by performing gene ontology (GO) term enrichment (Fig. 7F-H). The few DEGs at 14 days were associated primarily with apoptotic pathways and growth factor signaling. By 21 days, many dysregulated pathways emerged, including downregulation of mitochondrial metabolism genes and elevated expression of inflammatory pathways (wound response and apoptotic signaling), and dysregulation of intracellular signaling pathways. Finally, at the end stage, we found alterations in MAPK pathway genes and further dysregulation of mitochondrial pathways.

Lastly, we compared specific DEGs found with those described in a recent transcriptomic study that examined three centronuclear myopathy models, including *Mtm1* KO mice (Fig. 7I) (Djeddi et al., 2021). This comparison revealed eight consensus DEGs changed early in the disease process. For example, the transcriptional factor *Runx1* is upregulated at the earliest time point investigated, prior to observable muscle weakness. Additionally, *Tubb6*, which is a major component of the microtubule (Randazzo et al., 2019), was upregulated in all datasets, and at all time points in our study.

### Subcellular proteomics identifies changes in the proteosomal network and shows that the majority of changes occur in the organelle and membrane fraction

**Fig. 8.**
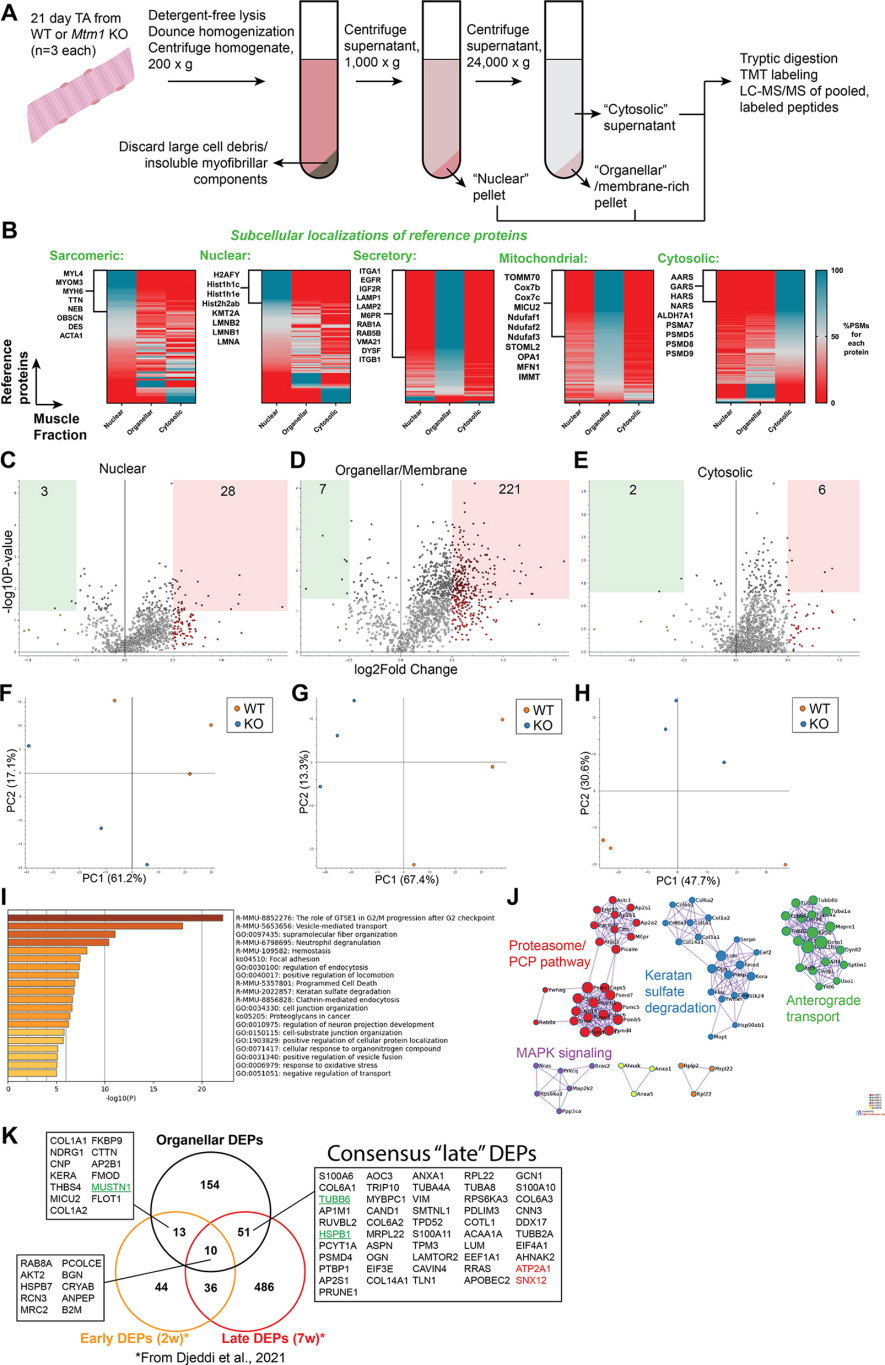
**Subcellular proteomics in *Mtm1* KO mice.** (A) Overview of methods used to perform proteomics on three subcellular fractions from 21-day-old mouse TA muscle. See Materials and Methods section for details. LC-MS/MS, liquid chromatography–tandem mass spectrometry; TMT, tandem mass tag. (B) Heat maps representing the enrichment of proteins in each of the muscle fractions with reference to consensus subcellular location data. Peptide spectrum matches (PSMs) provide an estimate of protein abundance and were used to show what percentage of PSMs for a given protein were identified in each fraction. Data are sorted based on which fraction had the greatest number of 100% PSMs for proteins in the reference set. (C-E) Volcano plots highlighting differentially expressed proteins (DEPs) in the nuclear (C), organellar/membrane (D) and cytosolic fractions (E) (*P*<0.05, log2FC>0.5). Statistical analysis by unpaired two-tailed Student's *t*-test. (F-H) Principal component (PC) analysis plots from each fraction. (I) GO term enrichment analysis of DEPs from the organellar/membrane fraction reveals dysregulation of focal adhesions and vesicle-mediated and endocytic pathways in *Mtm1* KO mice. (J) Protein–protein interaction network visualization of organellar fraction DEPs show aberrant levels of proteasomal proteins and MAPK signaling pathways in *Mtm1* KO mice. (K) Venn diagram highlighting DEPs found in common between the organellar fraction and those identified in a longitudinal study of *Mtm1* KO mice by Djeddi et al. (2021). Proteins in green were also identified as either early or late consensus DEGs. All DEPs shown had the same ‘sign’, i.e., upregulated versus downregulated, except for the two proteins indicated in red. PCP, planar cell polarity.

To corroborate our gene expression findings, and to better understand the subcellular relationship of the molecular changes, we performed proteomics on subcellular fractions of 21-day-old TA. We first confirmed the success of our fractionation methodology (Fig. 8A) by examining the expression of proteins known to localize to different compartments (Fig. 8B). In general, we saw good segregation between the five fractions generated. Notably, the nuclear fraction exhibited the most contamination by sarcomeric/myofibrillar components. We then explored differences between WT and *Mtm1* KO mice (Fig. 8C-H), and found the most significant differences in the membrane/organelle fraction, where >200 proteins were differentially expressed proteins (DEPs). Interrogation of pathways associated with these DEPs identified fiber organization, vesicle transport and focal adhesions as among the most significantly changed (Fig. 8I,J).

In subsequent network analyses, we observed changes in the ubiquitin–proteasome system and anterograde transport, among others (Fig. 8). Of note, membrane/organellar fraction were enriched in mitochondrial proteins. Given that mitochondrial abnormalities were observed at P21 by both histology and RNAseq, we focused on mitochondrial DEPs. We found elevated levels of MICU2, ATP5F1C, PDP1 and MRPL22, and decreased levels of NDUFA3, CYCS and MT-CYB, with the latter being important regulators of the mitochondrial respirometry complexes and suggestive of dysregulation of mitochondrial function (see below). Lastly, we highlight specific proteins that were also differentially expressed in a recent study using bulk proteomics in an *Mtm1* KO mouse model (Djeddi et al., 2021).

### MTM1 interacts with both MTMR10 and MTMR12 as well as several novel proteins

**Fig. 9.**
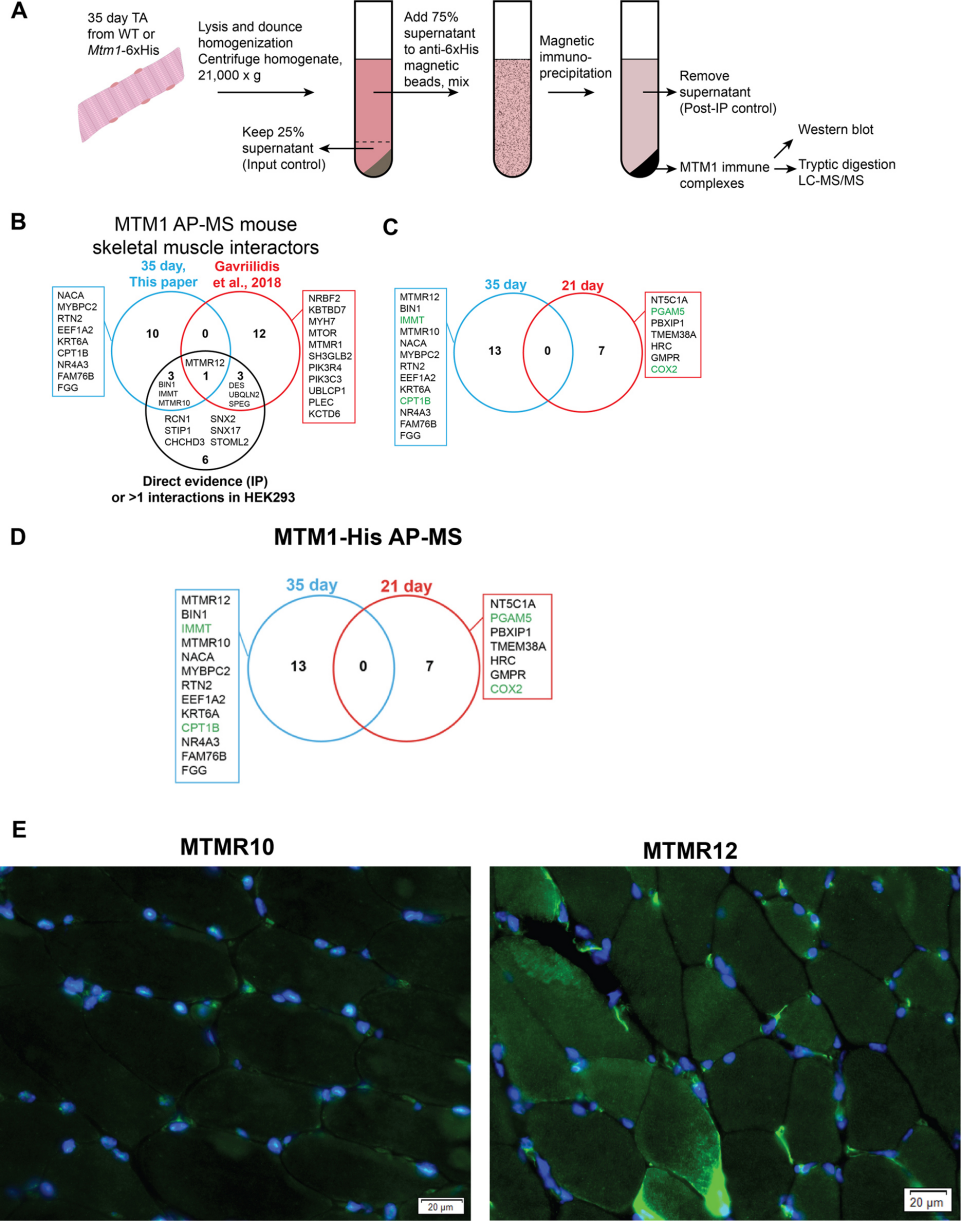
**MTM1 protein interactions in skeletal muscle at 21 and 35 days.** (A) Methodology for identifying putative MTM1 interactors using *Mtm1*-6xHis-tagged mice and immunoprecipitation (IP) followed by mass spectrometry. (B) Interactors identified from skeletal muscle extracts at 35 days, with comparison to interactors identified in previous studies. (C) Interactors from 21-day-old muscle extracts. Note no overlap with the proteins identified at 35 days. (D) Nuclear interactors identified at 35 days, including phosphatase-dead myotubularins MTMR10 and MTMR12. (E) Immunofluorescence images from antibody staining of cryosections from WT TA muscle. MTMR10 localizes primarily to the myonuclei (co-stained with DAPI), whereas MTMR12 is found primarily in the perinuclear compartment. Scale bars: 20 mm.

We next sought to identify relevant protein–protein interactions for MTM1 at different time points. To accomplish this, we used CRISPR/Cas9 gene editing to introduce a 6xHis tag in frame with the 3′ end of the mouse *Mtm1* gene. We then performed immunoprecipitation followed by mass spectrometry using anti-His beads (Fig. 9A). We started with muscle (TA) extracts from 35-day-old mice (Fig. 9B). We identified two known interactors, BIN1 and MTMR12, validating the ability of our methodology for finding MTM1-binding partners (Gupta et al., 2013; Royer et al., 2013). We also identified MTMR10, which, like MTMR12, is a phosphatase-dead member of the myotubularin family (Raess et al., 2017). Interestingly, MTMR10 localizes in skeletal muscle primarily in the myonuclei (Fig. 9D,E), suggesting the possibility that some MTM1 may be found outside of the endosome. This is also in line with other novel MTM1-binding partners identified in whole muscle extracts from this study, including reticulon 2 (RTN2) and nuclear receptor subfamily 4 group A member (NR4A3). We also identified IMMT, a mitochondrial membrane protein that is a member of the cristae-organizing system (MICOS) complex, which has been previously identified as an MTM1 interactor in two independent studies in HEK293 cells (https://thebiogrid.org/110630/summary/homo-sapiens/mtm1.html). We performed a similar analysis on muscle (TA) extracts from 21-day-old mice (Fig. 9C). Surprisingly, we found an essentially non-overlapping group of proteins, although several mitochondrial proteins were still identified, as well as multiple associations with regulators of nucleotide metabolism.

### Loss of *Mtm1* affects mitochondrial function

**Fig. 10.**
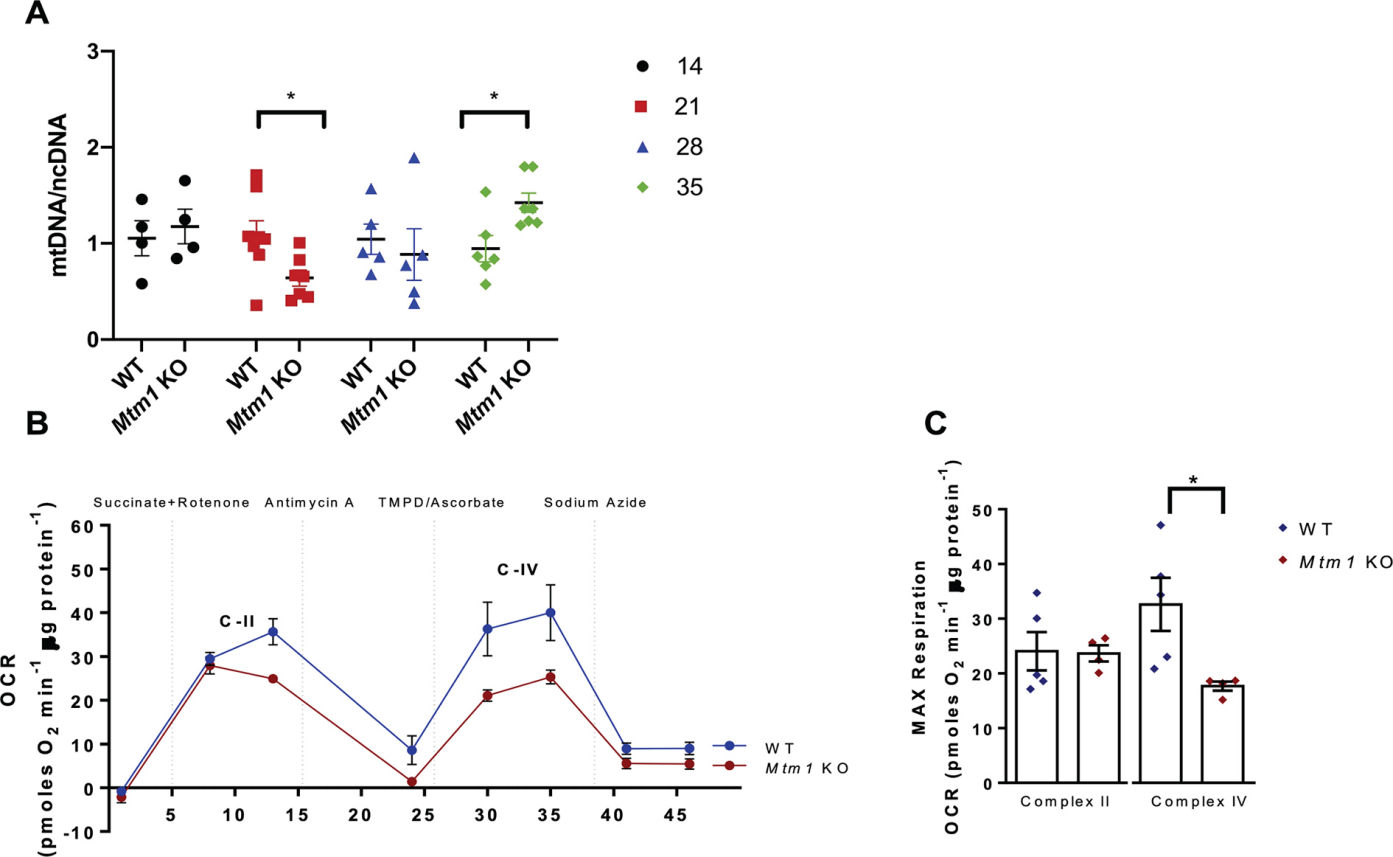
**Abnormal mitochondrial function in *Mtm1* KO mice.** (A) Mitochondrial DNA (mtDNA) to nuclear DNA (ncDNA) by quantitative RT-PCR in WT and *Mtm1* KO mice over time at 14, 21, 28 and 35 days. Values are mean±s.e.m. relative to WT at each time (WT *n*=4-7, KO *n*=4-7). (B,C) Respirometry in frozen muscle (RIFS), using the Seahorse bioanalyzer, done on mitochondria isolated from 35-day-old TA muscle. (B) Representative Seahorse traces of complex II- and IV-dependent respiration of WT and *Mtm1* KO mice. C-II, succinate+rotenone-induced respiration for complex II activity; C-IV, TMPD+ascorbate-induced respiration for complex IV activity. Values are mean±s.e.m. pmol O_2_/min adjusted per μg of protein. OCR, oxygen consumption rate. WT *n*=5; KO, *n*=4; values are provided as the mean±s.e.m. (C) Quantification of maximal respiration of complex II- and IV-dependent respiration (WT *n*=5, KO, *n*=4; values are mean±s.e.m.). Complex II: WT 24.06±5.39, KO 23.66±5.44; complex IV: WT 32.62±7.37, KO 17.69±4.02 (*P<0.05). Each biological replicate represents the average of two technical replicates. Statistical analysis by unpaired two-tailed Student's *t*-test.

Lastly, we aimed to determine whether mitochondrial dysregulation, as observed by our multi-omic datasets, equated to functional defects. We report high variability of mitochondrial DNA (mtDNA) copy number throughout the disease progression (Fig. 10A), with highest mtDNA copy number observed in *Mtm1* KO mice at 35 days. Notably, a positive relationship between mtDNA copy number and mitochondrial disease progression has been previously described (Filograna et al., 2021), likely as a compensatory mechanism. To provide insight into mitochondrial-mediated oxidative phosphorylation, we performed Seahorse analysis on isolated mitochondria from TA of *Mtm1* KO mice at P35 (Fig. 10B,C). This revealed specific defects related to complex IV, with significantly reduced maximal respiration in *Mtm1* KO compared to WT mice. This finding is line with our RNAseq and proteomic datasets, which identified several dysregulated targets related to complex IV activity. Together, these findings suggest an important interplay between MTM1, the mitochondria and disease pathogenesis, as supported by the observations of pathologic, molecular and functional changes with loss of *Mtm1*.

## DISCUSSION

**Fig. 11.**
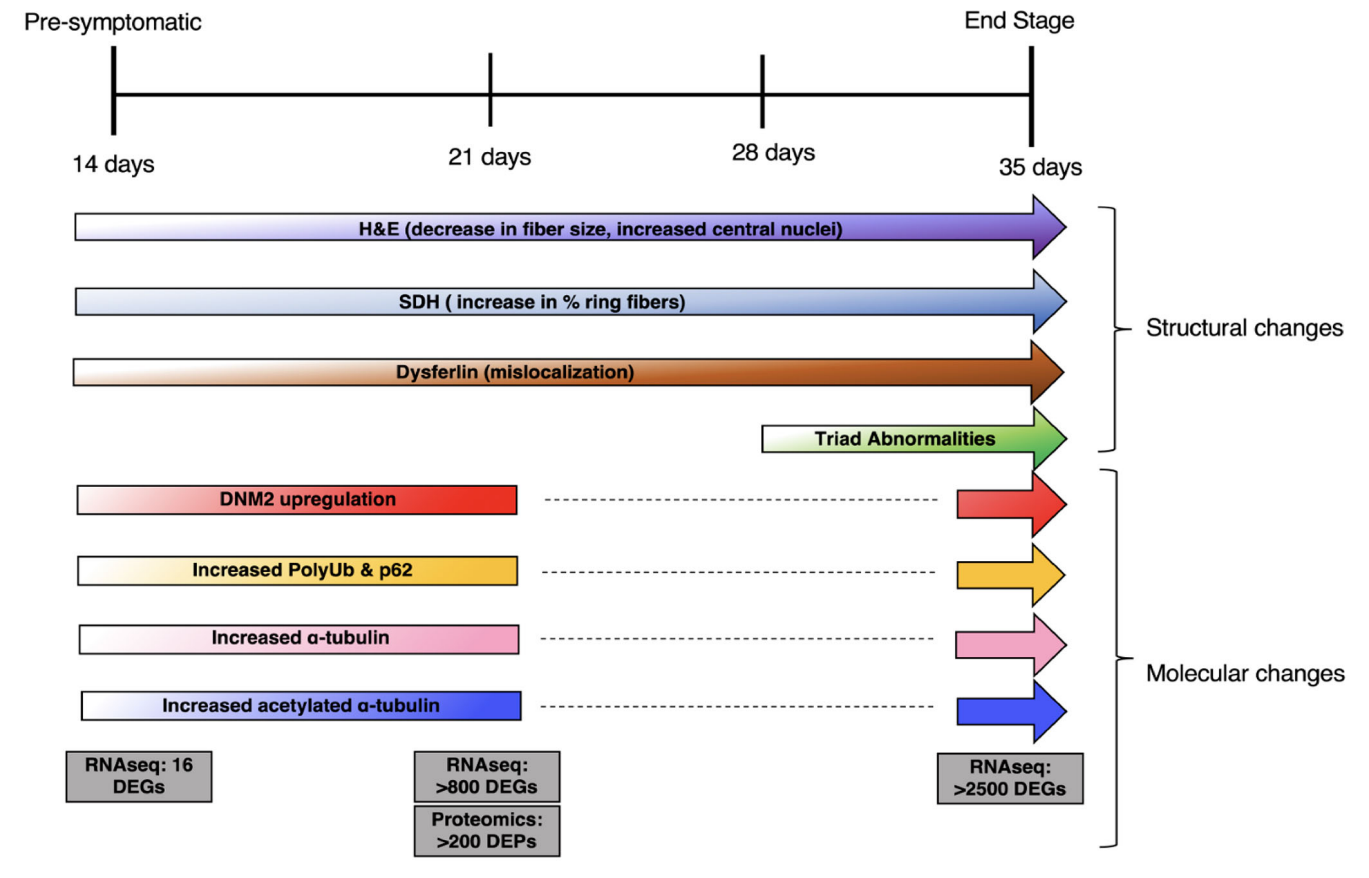
**Summary of pathologic and molecular changes in the *Mtm1* knockout mouse model.** Summary of the findings from this paper, including changes in histopathology and molecular markers, mapped across the four stages of the murine disease process. PolyUb, polyubiquitinated protein.

In this study, we present the natural history of a mouse model of XLMTM (Fig. 11). We identify molecular and structural changes in the muscle that precede the onset of overt phenotypic abnormalities, showing that the disease process occurs during muscle development and before the animal's present outward signs of weakness and muscle dysfunction. These early changes do not include alteration of triad structure, showing that triad formation occurs normally and suggesting that triad abnormalities are either a secondary consequence or the result of loss of MTM1 function in triad maintenance. These, and other observations, lay the groundwork for future experimentation aimed at uncovering the role of MTM1 in normal muscle development and elucidating the initiating steps in the XLMTM disease process. Finally, our data provide a guide for harmonization and uniformity of future pre-clinical interventional studies.

One of the most striking observations in our study is the early and pervasive change in mitochondrial distribution. This appears to be the first structural change in the muscle, and is consistent with the known interplay between MTM1 and desmin (Hnia et al., 2011), as the desmin intermediate filament network is known to be a key regulator of mitochondrial organization (Milner et al., 2000). MTM1/mitochondrial interplay may additionally be mediated by interactions between MTM1 and mitochondrial components that are required for optimal functioning of respiratory complexes. In this regard, it is notable that MTM1 interacts with IMMT, a component of the MICOS complex, which is required *in vitro* for optimal function of complex IV activity (Friedman et al., 2015), and that complex IV activity is reduced in P35 KO muscle. Given these data and known MTM1 function at the endosome, there is an intriguing possibility that MTM1 could be a key mediator of mitochondrial tethering, a process important for mitochondrial distribution and function, and controlled in part via interaction with the endosomal compartment (Elbaz-Alon et al., 2020). In terms of the XLMTM disease process, mitochondrial disorganization may contribute to changes in myofiber size and thus to the overall reduced body weight. It may also influence other molecular pathways that are abnormal later in the disease process, such as autophagy and the ubiquitin–proteasome system.

In contrast, triad changes do not occur until later in the disease process. They appear to coincide with overt severe muscle weakness, consistent with the hypothesis that they are a main driver of weakness in XLMTM. The interplay between MTM1 and the triad remains poorly understood. Our findings do not necessarily shed new light on this, except to support the existing idea that MTM1 is not required for triad formation, but rather for triad maintenance. How it governs triad maintenance is not clear. We confirm interaction with BIN1, a key protein for T-tubule biogenesis (Lee et al., 2002), and also the increase in DNM2 levels that occurs with loss of MTM1. Interruption of the MTM1–BIN1 interaction, or overexpression of DNM2, may be sufficient to explain how MTM1 influences the triad, although the role of BIN1–MTM1 binding still requires further study, and the mechanism(s) by which DNM2 protein levels (but not RNA levels) are increased in XLMTM is unknown. An interesting alternative consideration is that DNM2/BIN1/MTM1 participate in a membrane recycling process that utilizes the endosome and that governs triad membrane turnover. Such a turnover process has yet to be elucidated in skeletal muscle, but it fits with the cellular functions of these proteins. Finally, it is interesting to note that we did not detect association between MTM1 and BIN1 in our P21 mass spectrometry dataset, suggesting the potential that this association is present only at later time points that coincide with the alterations of triad structure.

We again demonstrate that DNM2 protein levels are increased at all time points tested and reveal, via RNAseq, that *Dnm2* transcripts are not increased. This supports the importance of DNM2 overexpression in the disease process and is consistent with the impressive impact of DNM2-lowering treatments on *Mtm1* KO mouse phenotypes (Cowling et al., 2014; Gayi et al., 2018; Maani et al., 2018; Tasfaout et al., 2017). In addition to DNM2 lowering, several other therapeutic strategies have been identified using the XLMTM mouse model. These include gene replacement therapy (Childers et al., 2014), enzyme replacement therapy (Lawlor et al., 2013), PIK3C2B lowering (Sabha et al., 2016) and pathway modifiers such as rapamycin (Fetalvero et al., 2013). Moving forward, it will be critical to have harmonization in the measures examined for existing treatments so that the field can best compare and contrast these therapies. Lack of uniformity in terms of techniques and outcomes has been identified as an important problem in pre-clinical therapy development and a key contributor to potential failures of translation for drugs in other rare diseases. We thus encourage use of standard sets of experiments and techniques when evaluating treatments in the *Mtm1* KO mouse model. From a general phenotypic perspective, body weight, open field gait analysis (particularly rearing behavior) and survival appear robust and reproducible. From a pathology perspective, mitochondrial organization, myofiber size and triad number are easily quantified, and reflect key aspects of the disease process. Molecular investigations should relate both to general disease pathway changes, such as DNM2 and polyubiquitin changes, and to the specific therapeutic to demonstrate clear target engagement.

One novel aspect of the study is the inclusion of muscle MRI. We show that the MRI findings correlate well with our analyses of myofiber size across the different muscle groups, with a similar degree of change observed for a given muscle group with MRI volume and fiber size within that muscle. Overall, we noted higher-magnitude differences between KO and WT muscles with predominantly fast (type II) fibers, and less change in those containing predominantly slow (type I) fibers. Although there are not such granular data in patients related to myofiber size, there is a growing appreciation of muscle MRI findings. In general, there is diffuse atrophy and fatty replacement across most muscle groups in XLMTM patients, and therefore it is difficult to make meaningful comparisons with our data. That stated, in the proximal leg muscles of patients, there appears to be a more prominent involvement of anterior compartment musculature (Cassandrini et al., 2017). We similarly see preferential change in the proximal anterior compartment in *Mtm1* KO mice, suggesting good correlation between mouse and patient MRI.

Lastly, our data highlight new potential avenues for investigation into XLMTM disease pathomechanisms. This is highlighted, for example, when considering our longitudinal transcriptomics and subcellular proteomics, particularly in comparison to previous multi-omic studies. For example, we identified increased expression of Tubb6 through both RNAseq and proteomics. This protein is of potential interest given that its persistent upregulation has been associated with disorganization of microtubule networks and return to a more immature developmental stage in skeletal muscle (Randazzo et al., 2019), both observations seen in XLMTM skeletal muscle.

Our studies were performed on the C57BL6/J background. The mice were re-derived from ones on a mixed background (C57BL6J/129) and have been maintained by backcrossing to the parent C57BL6/J strain every three to four generations. The data presented in this paper are from mice on the C57BL6/J for >20 generations, and thus considered to be on a pure C57BL6/J background. Although a direct comparison with the mice on the original 129PAS background (Buj-Bello et al., 2002) was not possible in this study, it is likely that there are phenotypic differences between our mice and those on the original background and/or on other mixed backgrounds. One example is survival, where our mice have a median survival of 38 days, and longer survival is noted in Buj-Bello et al. (2002) and in subsequent studies using derivates from these mice (Buj-Bello et al., 2002; Gayi et al., 2018; Tasfaout et al., 2017). Importantly, however, there is excellent concordance with key findings between our mice and data from lines used by other investigative teams, including structural pathology (i.e. the change in triads) (Al-Qusairi et al., 2009; Sabha et al., 2016), molecular changes (e.g. DNM2 increase and with comparative elements of the transcriptome) (Cowling et al., 2014; Djeddi et al., 2021; Gayi et al., 2018) and treatment response (e.g. improvement across strains with tamoxifen (Gayi et al., 2018; Maani et al., 2018).

On a final note, an independent study by Buono et al. appearing in this issue of Disease Models & Mechanisms describes the natural history of *Mtm1* KO mice with the same genetic alteration (exon 4 deletion) but on the 129SvPAS background (Buono et al., 2022). This study elegantly dissects the progression of the *Mtm1* KO model, with a particular emphasis on phenotypic characteristics including body weight, muscle strength and survival, as well as responsiveness and rate of change of these parameters with intervention. It highlights some of the small, but distinct, differences between *Mtm1* KO mice maintained on different backgrounds. It also complements well the data in this paper, as our study focused primarily on age-dependent progression of molecular abnormalities.

In summary, we present phenotypic, molecular, and cellular changes of an XLMTM mouse model at four stages of the murine disease process (Figs 1 and 11). Our data provide a roadmap for testing of future therapies and for elucidating disease pathomechanisms.

## MATERIALS AND METHODS

### Mouse husbandry and genotyping

Mice were housed at the Toronto Centre for Phenogenomics (Toronto, ON, Canada). The *Mtm1* KO line was originally generated at the Institute of Genetics, Molecular and Cellular Biology (IGBMC; Strasbourg, France) in the 129S2PAS background (Buj-Bello et al., 2002). The line was rederived onto the C57BL6J background at the Toronto Centre for Phenogenomics. To limit genetic drift, carrier female mice were backcrossed to the original C57BL6J strain every three to four generations. Animals were housed four per cage with an approximate *Mtm1* KO:WT ratio of 1:1. Mice were housed in Techniplast Greenline individually ventilated transparent cages (391×199×160 mm) and enriched with enviropacks, gnawing blocks, tubes and innodomes. Mice were maintained in a 12 h light-dark cycle (07:00 lights on) in a temperature (22°C)- and humidity-controlled room. Water was provided via an automated water system, and all mice had *ad libitum* access to water and food. Mice were maintained on a chow diet (Envigo Labs, 2918) providing 18% protein, 6.2% fat and 44.2% carbohydrates (with 14.7% neutral detergent fiber). Housing conditions were adapted to increasing severity as mice aged by placing food pellets on the cage floor and/or adding a gel-based diet to the cage floor (DietGel 76A with wheat protein). Gel diet components can be found on the manufacturer's website (Clear H2O; https://www.clearh2o.com/product/dietgel-76a-w-plant-based-protein/). Mice were euthanized for tissue harvest by carbon dioxide chamber followed by cervical dislocation. Muscles (TA, quadriceps, gastrocnemius and hamstring) were excised and weighed. All methods listed below, except for muscle MRI, were performed using the TA. Muscle MRI images were segmented using ITK-SNAP, a software used to segment 3D medical images (http://www.itksnap.org/pmwiki/pmwiki.php). All animal procedures were performed in compliance with the Animals for Research Act of Ontario and the Guidelines of the Canadian Council on Animal Care (AUP#22-0255H). Animal genotyping was performed using PCR-based methods. Exon 4 deletion in *Mtm1* was detected using the following primer set: Fwd, 5′-AATGTGTGCATGTTTGGACC-3′; Rev, 5′-ACAGTGATGCACAGAGAGGA-3′.

### Grip strength and open field test

Motor functional analysis was done at the Clinical Phenogenomics Core facility in the Toronto Centre for Phenogenomics. Grip strength was performed as per standard protocol (TREAT-NMD standard operating procedure; https://treat-nmd.org/sop/dmd_m-2-2-001/) using the Bioseb BIO-GS3. Open field testing was done on eight open field arenas (43.5 cm^2^) with three 16-beam infrared arrays (*x*-, *y*- and *z*-axes) using equipment from Med Associates Inc. Equipment also included sound-attenuating chambers (Chambers 1-8) with 250-275 lux LED and metal collection pans. Activity captured with this technology was analyzed using monitoring software from Med Associates Inc. For both tests, the same animals were tested at 14, 21, 28 and 35 days of age in a blinded fashion without the knowledge of genotype.

### Histology and immunostaining

Frozen muscle tissue was cut using a cryostat into 8 mm cross sections and then stained with Mayer's H&E or SDH. For indirect immunofluorescence (IF), frozen sections were blocked for 1 h, then incubated overnight at 4°C with anti-dysferlin antibody (ab124684, Abcam), anti-MTMR10 antibody (ORB185350, Cedarlane) or anti-MTMR12 antibody (GTX119163, GeneTex), diluted 1:100 in blocking solution. Secondary antibodies (Alexa Fluor 488, Invitrogen) were applied for 1 h at room temperature at 1:1000 dilution. Micrographs were captured with an Infinity1 camera (Lumenera Corp.) through an Olympus BX43 light microscope. Quantification of the number of centrally nucleated fibers, myofiber size, SDH staining and dysferlin IF was performed manually using ImageJ from H&E, SDH and dysferlin IF photographs taken at 20× magnification.

### Transmission electron microscopy

Muscles were fixed in 2% glutaraldehyde containing 0.1 M sodium cacodylate buffer overnight at 4°C, then taken to the Advanced Bioimaging Center (The Hospital for Sick Children, Toronto, ON, Canada). Sections 90 nm thick were cut on an RMC MT6000 ultramicrotome, stained with uranyl acetate and lead citrate, and viewed with an FEI Tecnai 20 microscope.

### Western blot analysis

Muscle was flash-frozen on dry ice and immediately stored at −80°C. Muscle was lysed with steel beads in TissueLyserII (Qiagen), and cells were lysed using cell scraper in the presence of RIPA buffer (supplemented with protease and phosphatase inhibitors) to extract protein. Samples were centrifuged at 12,700 ***g*** for 30 min at 4°C. A Pierce™ BCA protein assay kit (Thermo Fisher Scientific) was used to quantify the protein levels in the supernatant. Then, 30 µg protein per lane was prepared with 4× loading buffer supplemented with dithiothreitol. Samples were boiled for 5 min at 95°C with the exception of samples for RyR1 blots, which were heated at 37°C for 10 min. Samples were run on SDS-PAGE gels until necessary and then transferred onto polyvinylidene fluoride (PVDF) membranes by semi-dry transfer method. Following the transfer, the membranes were treated with total-protein REVERT stain and visualized by a LI-COR instrument at 700 nm (Fig. S2). Membranes were blocked with 3% bovine serum albumin for 1 h at room temperature and then incubated with primary antibody overnight at 4°C. Primary antibodies used were anti-DNM2 (1:1000; sc-166669, Santa Cruz Biotechnology), anti-polyubiquitinated protein (1:1000; 04-262, Sigma-Aldrich), p62 (1:1000; 5114S, Cell Signaling Technology), anti-acetylated α-tubulin (1:2000; T6793, Sigma-Aldrich), anti-α-tubulin (1:1000; 2144, Cell Signaling Technology) and anti-MTM1 (Clone 2827, Dr Jocelyn Laporte, IGBMC). Secondary antibodies used were horseradish peroxidase-conjugated goat anti-rabbit or goat anti-mouse IgG secondary antibody (1:5000; Bio-Rad). Blots were visualized using chemiluminescence (Clarity Max™ ECL, Bio-Rad or Western Lightning ECL Pro, PerkinElmer) in the Gel Doc™ XR with Gel Documentation System (Bio-Rad). Membranes were stripped using Restore Western Blot Stripping Buffer (Thermo Fisher Scientific). Total protein and band intensities were measured using Fiji, and relative expression of proteins was normalized to the total protein staining of the lane. Every blot included four biological replicates and was run in triplicates for technical replicates.

### RNAseq and differential expression analysis

RNA extraction, library preparation and RNAseq were performed as previously described (Maani et al., 2018). Briefly, RNA was extracted from WT (*n*=4) and *Mtm1* KO (*n*=4) muscle at 14, 21 and 35 days by a Qiagen Fibrous RNeasy kit in accordance with the manufacturer's protocol. A NEBNext^®^ Ultra™ II Directional RNA Library Prep Kit for Illumina was used for library preparation, and ∼1000 ng RNA was used as starting material. The cut-off for RNA integrity number (RIN) was 8. Sequencing was done at The Centre for Applied Genomics (TCAG; Norwich, UK) using Illumina HiSeq 2500. Paired end read sequencing was performed to achieve 62-67 million reads per sample. Reads were aligned to the reference genome (GRCm38/MM10 version of the *Mus musculus* genome) and transcriptome using STAR51 two-step alignment to generate a Binary Alignment Map (BAM) file. Coordinate sorted BAM files were used to quantify transcript abundance (count data) using HTSeq52. Raw read counts generated were used as input for differential gene expression analysis, carried out using both DESeq2 and edgeR R/Bioconductor packages. This was carried out in a pairwise manner between any two conditions that were considered. False discovery rate (FDR)-adjusted *P*-values from both edgeR and DESeq2 were used to determine genes that are significantly differentially expressed between the conditions tested. Metascape was used for GO term enrichment of DEGs between time points (Zhou et al., 2019). Finally, we compared our DEGs to those reported in similar mouse models previously (Djeddi et al., 2021).

### Subcellular proteomics

The methods used in sample preparation, namely choice of lysis buffers and centrifugation speeds, were based on previously published methods (Dimauro et al., 2012; Itzhak et al., 2016). First, 21-day-old muscle (*n*=3 per replicate) was cut into small sections over dry ice. Next, sections were homogenized in a glass douncer over ice containing detergent-free lysis buffer (250 mM sucrose, 50 mM HEPES buffer pH 7.4, 5 mM MgCl_2_, 10 mM ATP, protease and phosphatase inhibitor cocktails). Tissue was homogenized with ten strokes of a loose pestle followed by 20 strokes of a tight pestle. Samples were then maintained on ice for 30 min. Homogenates were decanted in fresh 2 ml tubes and vortexed at maximum speed for 10 s, and centrifuged at 200 ***g*** for 5 min to remove large cellular debris and insoluble myofibrillar components. The pellet was discarded, and supernatant (S1) was moved into fresh 2 ml tubes, vortexed at maximum speed for 10 s and centrifuged at 1000 ***g*** for 15 min at 4°C. Supernatant (S2) was moved into fresh 2 ml tubes, and the ‘Nuclear’ pellet (P1) was placed on dry ice. S2 was vortexed at maximum speed for 10 s and centrifuged at 800 ***g*** for 10 min at 4°C to remove remaining/contaminating nuclei. Supernatant (S3) was moved into fresh 50 ml Nalgene round-bottom centrifuge tubes, and the pellet (P2) containing residual nuclei was discarded. S3 was vortexed at maximum speed for 10 s and centrifuged at 24,000 ***g*** for 20 min at 4°C. ‘Cytosolic’ supernatant (S4) was moved into new 2 ml conical tubes and placed on dry ice. Finally, the ‘Organellar/Membrane’ pellet (P3) was placed on dry ice.

Lysis buffer (RIPA buffer+1% SDS+protease and phosphatase inhibitors) was used to re-suspend pellets P1 and P3, heated to 72°C for 5 min with intermittent vortexing. Re-suspended pellets and cytosolic fraction S3 were sonicated for ten cycles (30 s on/off) using a Diagenode Bioruptor at high setting and at 4°C. Nuclear samples (P1) were treated with 25 U/ml benzonase endonuclease at room temperature for 30 min. Protein concentrations of all samples were quantified using the BCA protein assay before being sent for proteomics analysis. Additional sample preparation and mass spectrometry analysis were performed by the Network Biology Collaborative Centre (NBCC) at Lunenfeld-Tanenbaum Research Institute (Toronto, ON, Canada). One-hundred micrograms of protein were processed from each sample using S-traps (ProtiFi) as per the manufacturer's protocols and digested with 5 µg of trypsin each. Next, 10 µg of each sample was labeled with 80 µg of TMT10plex™ Isobaric Label Reagent (ThermoFisher Scientific, cat. 90406) per manufacturer protocols. For each subcellular fractionation (nucleus, membrane, cytoplasm), we used *n*=3 biological replicates from WT or *Mtm1* KO mutants. Each subcellular fraction was multiplexed with six TMT labels for three independent 6-plex experiments. Trapped and trypsin-digested peptides were acquired on a 180 min gradient using an Orbitrap Fusion Lumos Tribrid mass spectrometer. Data were searched with SequestHT using the UP000000589 proteome (*Mus musculus*) generated by UniProt, post-processed with Percolator and analyzed using Proteome Discoverer 2.2.0.388 (Thermo Fisher Scientific). Metascape was used for GO term enrichment and protein–protein interaction network analysis (Zhou et al., 2019). To test whether or not the obtained fractions were enriched in expected proteins, we cross-referenced identified proteins with a resource database that predicts subcellular location of proteins based on mass spectrometry data (Orre et al., 2019). Peptide spectral matches (PSMs) were used as a proxy for relative abundance given that PSMs indicate how many times a peptide of the particular protein was selected for MS2 fragmentation and confidently identified. The percentage of PSMs for a given protein in each fraction was calculated over the total number of PSMs for that protein. In this way, we could visualize how often that peptide was identified in one fraction relative to the others to estimate the degree of our subcellular enrichment.

### Immunoprecipitation–mass spectrometry (IP-MS)

Muscle was harvested from 21-day-old (*n*=3/genotype) and 35-day-old (*n*=2/genotype) WT and *Mtm1*-6xHis mice and stored at −80°C. Frozen muscle samples (∼30 mg) were minced and lysed in lysis buffer [50 mM HEPES-NaOH pH 8.0, 100 mM KCl, 0.1% NP40 and 10% glycerol, supplemented with 1 mM PMSF, 1 mM TCEP and 1× protease inhibitor cocktail (Sigma-Aldrich)] using a dounce tissue grinder (357544, Wheaton Science). Samples were centrifuged at maximum speed (18,213 ***g***) for 30 min at 4°C, and supernatants were collected. Magnetic dynabeads (10001D, Thermo Fisher Scientific) were washed in PBS and incubated with 4 μg anti-6xHis antibody (05-949, Sigma-Aldrich) overnight at 4°C with gentle agitation. Supernatants were incubated with 25 μl pre-conjugated beads for 2 h at 4°C with gentle agitation. Following this incubation, beads were washed once with lysis buffer, then washed twice with wash buffer [20 mM Tris-HCl (pH 8.0) and 2 mM CaCl_2_]. Beads were submitted to the NBCC, where samples were further washed, trypsin digested and eluted off the beads prior to liquid chromatography–mass spectrometry. SAINT analysis was performed by the facility.

### Statistical analysis

All data presented are expressed as mean±s.e.m. (unless otherwise specified) and were analyzed for statistical significance by unpaired two-tailed Student's *t*-test (unless stated otherwise). *P*<0.05 was considered to be statistically significant. GraphPad Prism software, version 8.0 for was used for generating graphs and performing statistical analysis.

### mtDNA:nuclear DNA (ncDNA) ratio

mtDNA copy number was calculated by determining the ratio of mtDNA:ncDNA via quantitative PCR as previously described. The primers used were as follows: mt-TL1: Fwd, 5′-GGTTCAAATCCTCTCCCTAATAGTGT-3′; Rev, 5′-TTAGGGCCTTTTCGTAGTTGTATGTAC-3′; complex IV (nuclear encoded): Fwd, 5′-GAGAAAGTTCAGTGTGGGTATCA-3′; Rev, 5′-CCAGCAGTATGGAATGGGTATC-3′.

### Respirometry in frozen samples (RIFS)

Respirometry of previously snap-frozen TA mitochondria isolates were measured in MAS buffer following the RIFS protocol as previously described, with minor modifications (Acin-Perez et al., 2020). Briefly, 20 mg sample was thawed in ice-cold PBS and homogenized in 1× MAS buffer using a glass-Teflon dounce homogenizer and incubated in collagenase type II (0.25 mg/ml final) in MAS at 37°C for 30 min. Samples were first centrifuged for 5 min at 1000 ***g***, 4°C (without a break) and then the supernatant was centrifuged for 10 min at 10,000 ***g***, 4°C (no break). The mitochondrial pellet was washed once and re-suspended in a final volume of 100 µl ice-cold MAS followed by a BCA protein assay to normalize the sample for loading. For bioenergic analyses, 6 µg of mitochondria isolates was loaded into a Seahorse XFe24 microplate in 30 µl MAS buffer and centrifuged for 5 min at 2000 ***g***, 4°C (no break) to ensure even distribution of mitochondria. Then, 120 µl of 1× MAS was carefully added per well. The cartridge was loaded to deliver 25 µl of the following injections (pH 7.2): Port A: 5 mM succinate+2 µM rotenone (complex II substrate+complex I inhibitor); Port B: 4 µM antimycin A (complex III inhibitor); Port C: 0.5 mM N,N,N′,N′-tetramethyl-p-phenylenediamine (TMPD)+1 mM ascorbic acid (electron donor to cytochrome complex IV+stabilizer); and Port D: 50 mM sodium azide (complex IV inhibitor). The Seahorse XFe24 Analyzer was generously provided by the Beaudry laboratory at the University of Toronto. For calculation of maximal oxygen consumption rate (OCR), the following equations were used: complex II (CII): OCR_CII_ – OCR_antimycin A_; and complex IV: OCR_TMPD+ascorbic acid_ – OCR_sodium azide_.

## Supplementary Material

Supplementary information
