# Supplementary material for: Natural history of a mouse model of X-linked myotubular myopathy
Source: Dis Model Mech. 2022 Jul 25;15(7):dmm049342. doi: 10.1242/dmm.049342 (PMC9346535; doi:10.1242/dmm.049342)
Supplement: Supplementary information [file dmm-15-049342-s1.pdf]

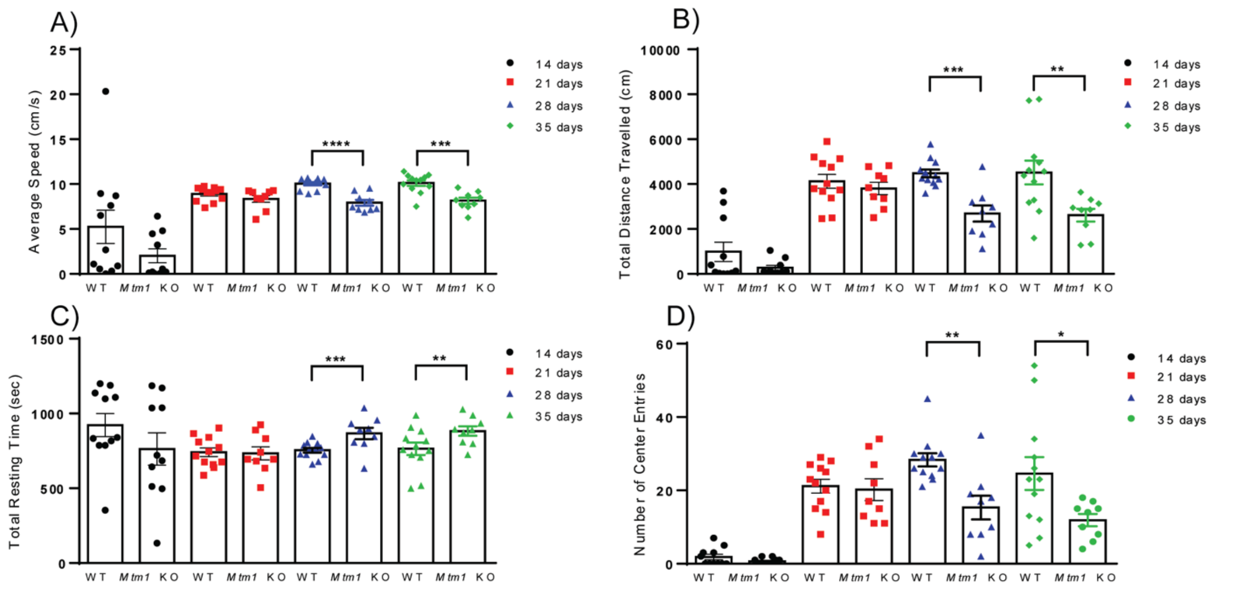

Fig. S1. Open Field Test Parameters in WT vs Mtm1 KO mice at 14, 21, 28 and 35days. *Mtm1* KO mice have reduced locomotor activity and increased resting time compared to WT mice at 28 and 35 days. All values are Mean ± SEM. (WT n=12, KO n= 9). **(A)** Average Speed (cm/s) travelled in whole arena at 14 days: WT 5.24 ± 1.85, KO 2.0 ± 0.7 ; 21 days: WT 8.9 ± 0.23 , KO 8.34 ± 0.37 ; 28 days: WT 10.04 ± 0.18 , KO 7.92 ± 0.33 (cm/s) (****p < 0.0001); 35 days: WT 10.1 ± 0.31 , KO 8.14 ± 0.34 (***p < 0.001). **(B)** Total Distance Traveled (cm) in whole arena at 14 days: WT 984.9 ± 427.28, KO 272.3 ± 100.27; 21 days: WT 4121.54 ± 310.6 , KO 3803.89 ± 273.64 ; 28 days: WT 4475.15 ± 171.97 , KO 2684.96 ± 359.82 (***p < 0.001); 35 days: WT 4515.96 ± 526.67 , KO 2612.13 ± 280.06 (**p < 0.01). **(C)** Total resting time (sec) in whole arena at 14 days: WT 922.4 ± 76.5 , KO 762.3 ± 115.77 ; 21 days: WT 740.46 ± 28.61 , KO 732.7± 43.9 ; 28 days: WT 754.04 ± 15.47 , KO 866.62 ± 38.33 (***p < 0.001); 35 days: WT 764.03 ± 41.54 , KO 882.14 ± 31.77 (**p < 0.01). **(D)** Total number of center entries at 14 days: WT 1.8 ± 0.74, KO 0.70 ± 0.24; 21 days: WT 21.16 ± 1.88, KO 20.22 ± 0.78; 28 days: WT 28.33 ± 1.79, KO 15.33 ± 3.24 (**p<0.01); 35 days: WT 24.58 ± 4.47, KO 11.88 ± 1.68 (*p<0.05). Data was analyzed by unpaired Student T-test.

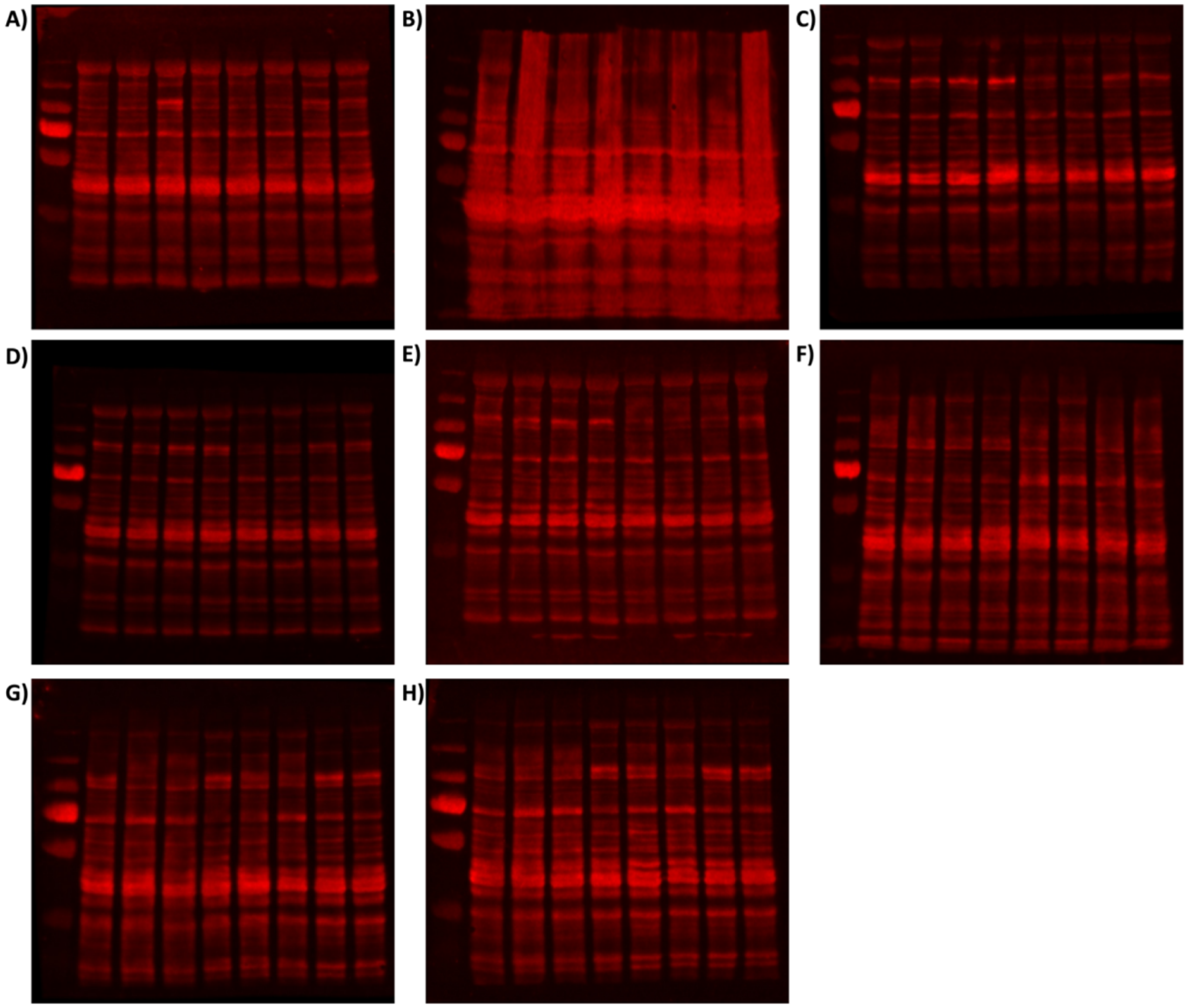

Fig. S2. REVERT total protein stains of the membranes from Figure 5. (A) 14 day DNM2, polyubiquitinated proteins, p62, alpha-tubulin and acetylated-alpha-tubulin blots. **(B)** 21 day DNM2. **(C)** 21 day alpha-tubulin and acetylated-alpha-tubulin blots. **(D)** 21 day polyubiquitinated protein blot. **(E)** 21 day p62 blot. **(F)** 35 day DNM2 blot. **(G)** 35 day p62 blot. **(H)** 35 day polyubiquitinated protein, alpha-tubulin and acetylated-alpha-tubulin blots. Membranes were stained with REVERT total protein stain to visualize total protein extract loaded on each lane and imaged at 700nm using LI-COR Odyssey Fc Imager.
